# Long-lasting reflexive and nonreflexive pain responses in two mouse models of fibromyalgia-like condition

**DOI:** 10.1038/s41598-022-13968-7

**Published:** 2022-06-12

**Authors:** Beltrán Álvarez-Pérez, Meritxell Deulofeu, Judit Homs, Manuel Merlos, José Miguel Vela, Enrique Verdú, Pere Boadas-Vaello

**Affiliations:** 1 Research Group of Clinical Anatomy, Embryology and Neuroscience (NEOMA), Department of Medical Sciences, Facultat de Medicina, Universitat de Girona (UdG), Emili Grahit 77, 17003 Girona, Catalonia Spain; 2 University School of Health and Sport (EUSES), University of Girona, Girona, Catalonia Spain; 3 WeLab Barcelona, Parc Científic de Barcelona, Barcelona, Catalonia Spain

**Keywords:** Experimental models of disease, Preclinical research, Chronic pain

## Abstract

Nociplastic pain arises from altered nociception despite no clear evidence of tissue or somatosensory system damage, and fibromyalgia syndrome can be highlighted as a prototype of this chronic pain subtype. Currently, there is a lack of effective treatments to alleviate both reflexive and nonreflexive pain responses associated with fibromyalgia condition, and suitable preclinical models are needed to assess new pharmacological strategies. In this context, although in recent years some remarkable animal models have been developed to mimic the main characteristics of human fibromyalgia, most of them show pain responses in the short term. Considering the chronicity of this condition, the present work aimed to develop two mouse models showing long-lasting reflexive and nonreflexive pain responses after several reserpine (RIM) or intramuscular acid saline solution (ASI) injections. To our knowledge, this is the first study showing that RIM6 and ASI mouse models show reflexive and nonreflexive responses up to 5–6 weeks, accompanied by either astro- or microgliosis in the spinal cord as pivotal physiopathology processes related to such condition development. In addition, acute treatment with pregabalin resulted in reflexive pain response alleviation in both the RIM6 and ASI models. Consequently, both may be considered suitable experimental models of fibromyalgia-like condition, especially RIM6.

## Introduction

Chronic or pathological pain represents long-term pain that persists even after the disappearance of the potential noxious stimulus or injury healing for more than 3 months^1^ and is the result of a malfunction of the somatosensory system^2^. In general, pathological pain may be classified into pain mechanism categories (PMCs) based on the characteristics of their presentation^3^. The International Association for the Study of Pain (IASP) classifies pathological pain into three main PMCs: (1) nociceptive, arising from actual or threatened damage to non-neuronal tissues; (2) neuropathic, which is caused by a lesion or disease of the somatosensory nervous system; and (3) nociplastic, due to altered nociception despite no clear evidence of actual or threatened tissue damage^1,4^. The precise mechanisms underlying nociplastic pain, the newly recognized third pain category, remain unknown^5^. However, it has been suggested that augmented CNS pain and sensory processing and altered pain modulation play prominent roles in nociplastic pain^5^.

Several health disorders involve nociplastic components^6,7^. Among them, fibromyalgia syndrome (FMS) is highlighted as a prototypical nociplastic pain condition^8^. Nociplastic pain rarely occurs in isolation and is usually accompanied by other CNS-associated symptoms, such as fatigue, cognitive impairment, hypersensitivity to external stimuli, and mood disturbances (especially depression and anxiety), classically described in FMS^5,8–10^. Although the prevalence of FMS is highly dependent on the case-finding detection methods and the diagnostic criteria, the worldwide prevalence of FMS is estimated to be approximately 2–4%^5^ in the general population, with a 3:1 sex ratio (female:male)^11^. Despite this prevalence, FMS continues to be an unmet need as far as pharmacological treatment is concerned since treatment options for FMS remain insufficiently effective^12^. Currently, the efficacy of current pharmacological strategies^13^ for nociplastic pain management remains lamentably low, and FMS patients tend to be extremely sensitive to drug side effects^12^. It is worth mentioning that some pharmacological treatments provide clinically significant improvement without major adverse events for a relatively small subset of patients, but in many others, the adverse effects overshadow the benefits or do not result in any symptom improvement^14^. This variability in response to drugs should not be surprising since the underlying mechanisms of FMS are still poorly understood^7,14^ and may differ between patients^14^.

There is a lack of efficient treatments to alleviate nociplastic pain, and new pharmacological strategies need to be developed to properly relieve pain in FMS patients. To this end, preclinical experimental models mimicking the main characteristics of human FMS, including long-lasting nociplastic pain and mood disorders (e.g., anxiety, depression), not primarily induced by traumatic injury or tissue damage, are needed. One of the animal models that reproduces most of the FMS signs and symptoms observed in humans is reserpine-induced myalgia (RIM)^15,16^. Available RIM models manifest widespread pain and hyperalgesia without apparent tissue damage^15,16^, an increased proportion of activated microglial cells in the spinal dorsal horn^17^, and molecular changes (e.g., increased substance-P, TNF-alpha, lipid peroxide, and elevated expression of GluN2B-NMDA receptors) in supraspinal structures^18,19^. This model consists of repeated administration of reserpine to patterns of 3 (RIM3)^15–21^, 4 (RIM4)^16^ and 6 (RIM6)^16^ administrations, in which biogenic amine depletion may cause muscular pain and tactile allodynia accompanied by depressive-like behaviors. Intramuscular acid saline solution (ASI) injection is also an experimental model that induces hyperalgesia without apparent tissue damage^22^. In this experimental model, skin and muscle mechanical hyperalgesia may be observed for 1–4 weeks after peripheral administration (gastrocnemius muscle)^22–27^, accompanied occasionally by anxiety and depression^27^. In this case, in contrast to RIM models in which biogenic depletion might affect the CNS pain pathways, in the ASI model, the aim is to generate central pathological pain after a peripheral trigger. Although these models are available, most studies have been performed in the short term. Considering the chronicity of FMS, long-lasting experimental studies are needed to better approximate human reality and improve the translational value of pharmacological studies. Moreover, there is little information on pathophysiological processes associated with both models, the pivotal role of central sensitization processes^7,12^ and the role played by long-lasting CNS glia activation in RIM and ASI models. The present work aimed to evaluate, over the long term, the time course of reflexive pain responses (thermal hyperalgesia and mechanical allodynia) and spinal cord gliosis in RIM3, RIM4, RIM6 and ASI mouse models, as well as nonreflexive pain responses (anxiety- and depression-like behaviors). Additionally, an acute dose–response study following pregabalin administration was performed in both models to evaluate whether this FDA-approved^12,13^ drug for FMS treatment exerts beneficial effects on FMS-like models.

## Results

### General observations

Following a protocol animal welfare supervision based on Morton D.B and Griffiths P.H. guidelines^28^, changes in coat and skin, vibrissae of nose, nasal secretions, signs of autotomy of hindpaw and/or forepaw, or aggressiveness were not detected in mice after either reserpine or acidified solution administration at any time of the experimental period. Moreover, the animals showed no significant weight loss throughout the experiment, which would also be related to either distress or system toxicity. Concretely, regarding the mice body weight, the MANOVA analysis of RIM models’ data indicated significant effects in the day factor (*p* < 0.001) but a lack of significance of both induction (*p* = 0.080) and interaction for day × induction (*p* = 0.270) factors (see Supplementary Fig. S1A). Similarly, the MANOVA analysis of ASI mice weight indicated significant effects in the day factor (*p* < 0.001) but a lack of significance of both induction (*p* = 0.416) and interaction for day × induction (*p* = 0.519) factors (see Supplementary Fig. S1B). Thus, these results indicate that neither reserpine nor acidified solution administration result in weight-loss. Actually, these results indicate that there is an increase of body weight during the treatment period but such increase effects equally to all of experimental groups, including controls.

### Long-lasting reflexive pain responses in RIM3, RIM4, RIM6 and ASI animal models

As mentioned, long-lasting reflexive pain responses to thermal and mechanical stimuli in either ASI or RIM mouse models remain unknown. We evaluated and compared the paw withdrawal response to thermal and mechanical stimulation of the ASI, RIM3, RIM4 and RIM6 models up to 6 weeks after the first administration of reserpine or acidified saline solution.

**Figure 1:**
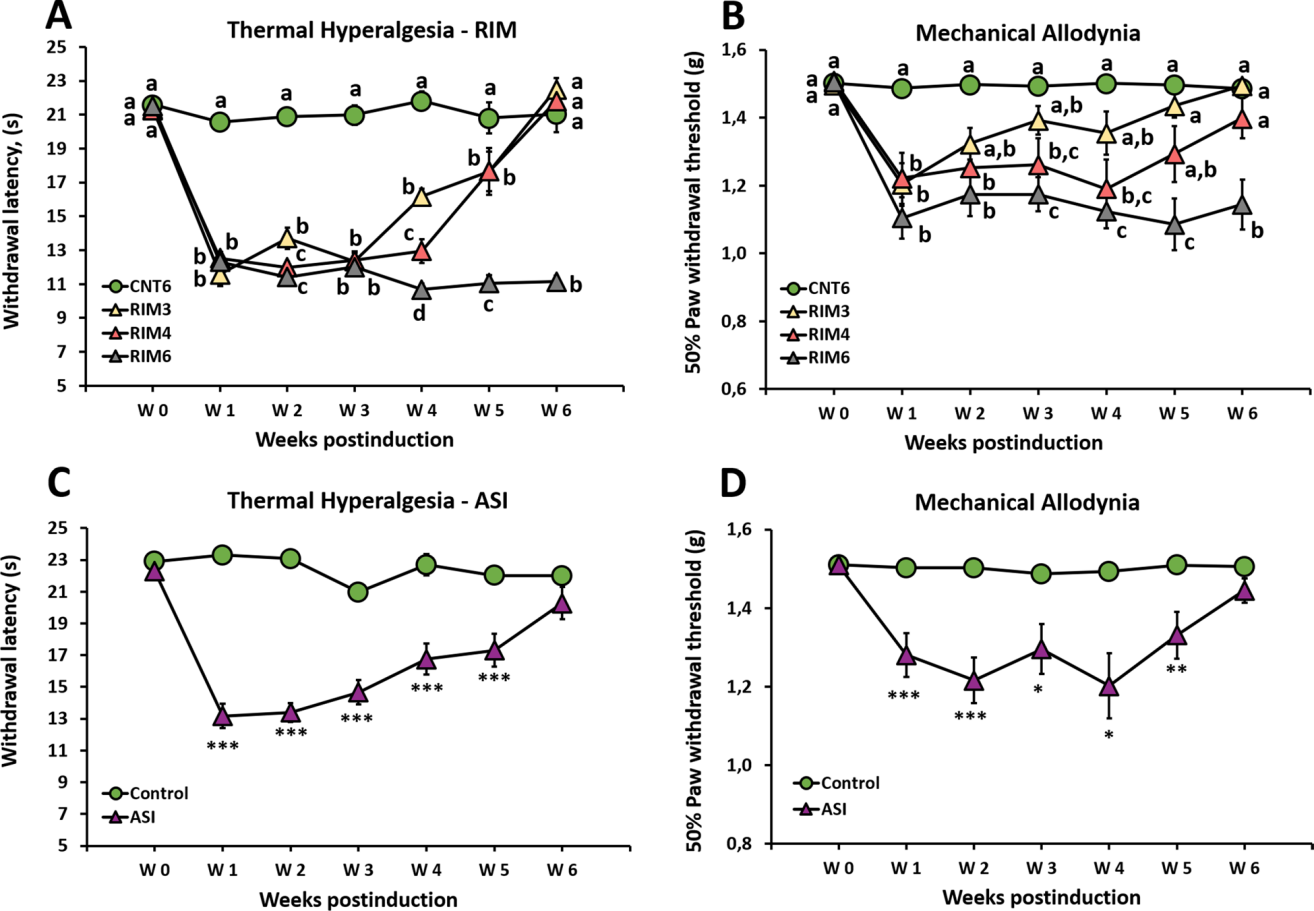
Time course of reflexive pain responses in mice subjected to reserpine-induced myalgia and intramuscular acid saline solution injection. The RIM6 mouse model shows longer-lasting reflexive pain responses than either RIM3 or RIM4 since only RIM6 animals showed significant thermal hyperalgesia (**A**) and mechanical hypersensitivity (**B**) up to 6 weeks postinduction. The ASI mouse model shows long-lasting thermal hyperalgesia (**C**) and mechanical hypersensitivity (**D**) up to 5 weeks postinduction. Each point and vertical line represent the mean ± standard error of the mean (n = 9–12 per group). a–d: Groups not sharing a letter are significantly different, *p* < 0.05 according to post hoc test; ***p < 0.0001, **p < 0.01, *p < 0.05 significant decrease versus ASI-Control, by post hoc tests.

Regarding thermal hyperalgesia in the RIM models, the data distribution significantly varied during the experimental period (Friedman test; *p* < 0.001), and significant group differences were observed from postinduction weeks 1 to 6 (all *p* values < 0.001; Kruskal–Wallis test). Specifically, the withdrawal latency to thermal stimulation was significantly lower in reserpine-induced myalgia mice (RIM3, RIM4, RIM6) than in control animals (CNT6) during the first three postinduction weeks (all *p*’s < 0.001; Mann–Whitney U-test), without major differences between them (Fig. 1A). In weeks 4 and 5, both the RIM3 and RIM4 models showed significantly decreased withdrawal latency to thermal stimulation in comparison to CNT6 (all *p*’s < 0.005), and RIM6 showed the significantly lowest withdrawal latency (all *p*’s < 0.005 vs. CNT6; RIM3 and RIM4 *p*’s < 0.002 vs. RIM6). Finally, at the end of the experimental period (week 6), significant thermal hyperalgesia was observed in RIM6 (all *p*’s < 0.001 vs. CNT6, RIM3, RIM4) but not in RIM3 and RIM4 (all *p*’s > 0.005 vs. CNT6) (Fig. 1A). Accordingly, the RIM6 mouse model showed longer-lasting (up to 6 weeks postinduction) thermal hyperalgesia than either RIM3 or RIM4. On the other hand, the thermal hyperalgesia after intramuscular injections of acidified saline solution significantly varied during the experimental period (Friedman test; *p* < 0.001) (Fig. 1C). Thermal hyperalgesia was maximal at week 1 and decreased over time, but withdrawal latencies to thermal stimulation remained significantly lower in ASI mice than in controls up to 5 weeks postinduction (all *p*’s < 0.005; Mann–Whitney U-test). Thus, thermal reflexive pain responses lasted up to 5 weeks postinduction in the RIM3, RIM4 and ASI models and up to 6 weeks in the RIM6 model.

Similar results were obtained in the reserpine-induced myalgia model with regard to mechanical allodynia, although differences with respect to controls were lower than in thermal hyperalgesia. The data distribution significantly varied during the experimental period (*p* < 0.001; Friedman test), and significant group differences were found from postinduction weeks 1 to 6 (all *p*’s < 0.002; Kruskal–Wallis test) in the RIM models (Fig. 1B). One week postinduction, RIM3, RIM4 and RIM6 showed a significant decrease in paw withdrawal mechanical thresholds when compared with CNT6 (all *p*’s < 0.005; Mann–Whitney U-test). Thereafter, this reflexive pain response decreased over subsequent weeks in RIM3 and RIM4 mice, with no significant difference from controls since weeks 2 and 5, respectively. In the case of RIM6, mechanical hypersensitivity was significant up to week 6 postinduction when compared with CNT6 (all *p*’s < 0.001) (Fig. 1B). Thus, the RIM6 mouse model showed longer-lasting (up to 6 weeks postinduction) mechanical hypersensitivity than either RIM3 or RIM4. Similar to the RIM model, the mechanical sensitivity data distribution also significantly varied during the experimental period (*p* < 0.0001; Friedman test) (Fig. 1D) in the ASI model. ASI-induced mechanical hypersensitivity also decreased over time, and the paw withdrawal mechanical thresholds were significantly lower in ASI mice than in controls up to 5 weeks postinduction (all *p*’s < 0.040, week 6 *p* = 0.091; Mann–Whitney U-test) (Fig. 1D).

### Spinal cord gliosis after repeated administration of either subcutaneous reserpine or intramuscular acidified saline solution injections

**Figure 2:**
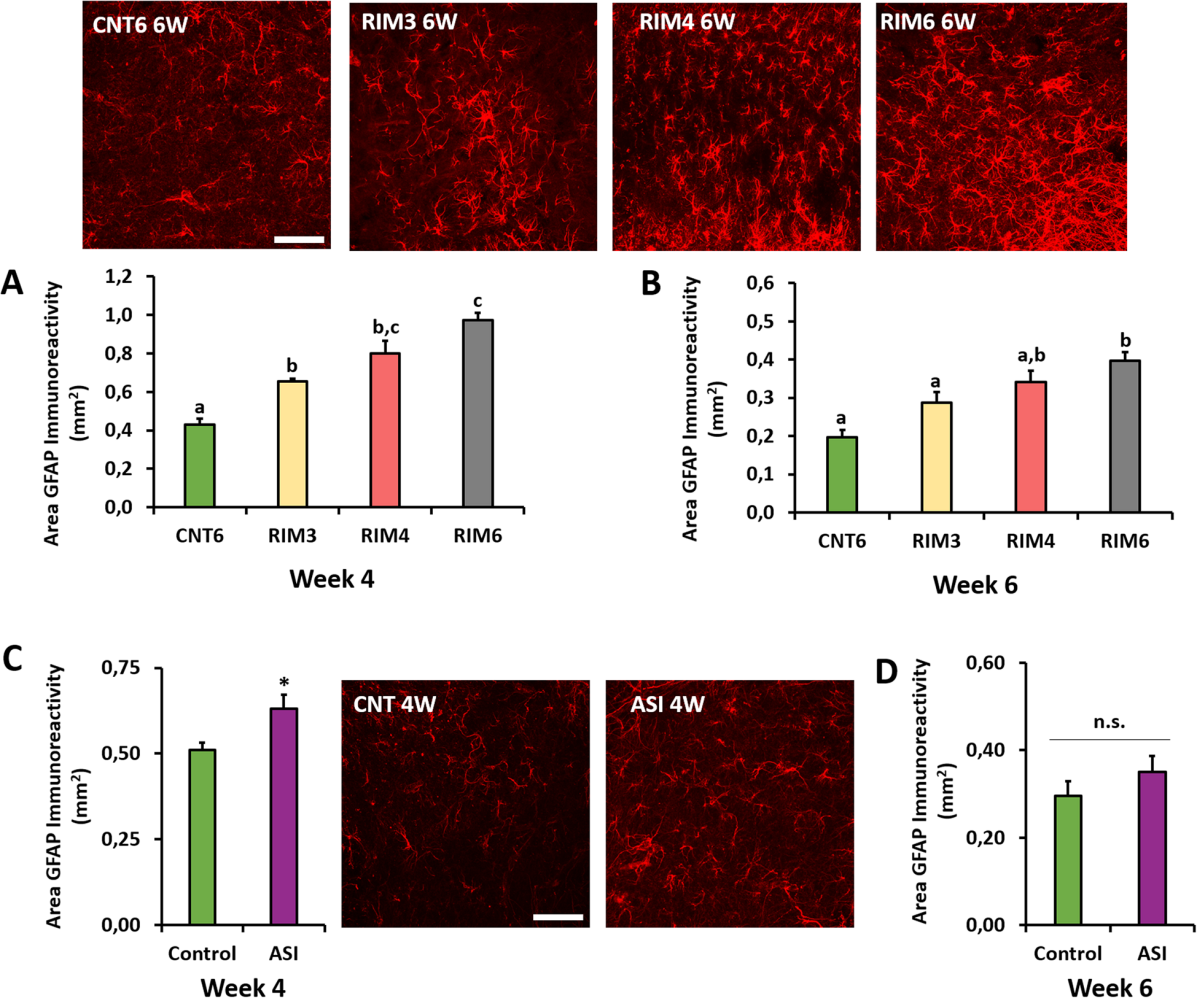
Spinal cord astrogliosis in mice subjected to reserpine-induced myalgia and intramuscular acid saline solution injection. Representative histological images of GFAP-positive cells are presented around the figure. Histograms show the percentage of dorsal horn GFAP immunoreactivity in the RIM groups at **(A)** four and **(B)** six weeks postinduction and ASI after **(C)** four and **(D)** six weeks. Data are expressed as the mean ± SEM. a–c: Groups not sharing a letter showed significant differences, *p* < 0.05; *p < 0.05 significant decrease versus ASI-Control, by post hoc tests.

**Figure 3:**
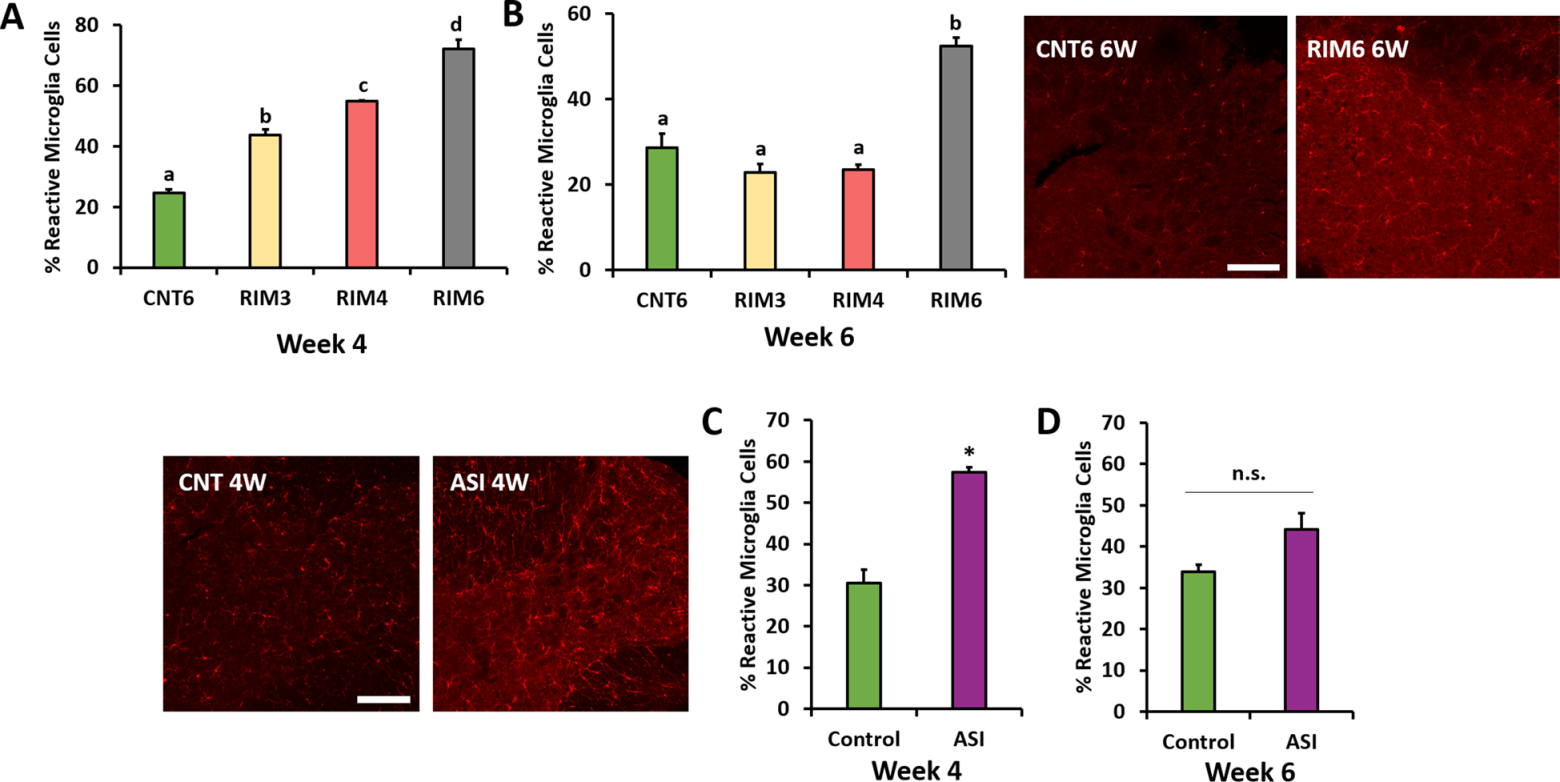
Spinal cord microgliosis in mice subjected to reserpine-induced myalgia and intramuscular acid saline solution injection. Representative histological images of IBA1-positive cells are presented around the figure. Reactive microglial cells showed shorter cytoplasmic extensions and an ameboid shape. Histograms show the percentage of reactive microglial cells in the RIM groups at **(A)** four and **(B)** six weeks postinduction and ASI after **(C)** four and **(D)** six weeks. Data are expressed as the mean ± SEM. a–d: Groups not sharing a letter showed significant differences, *p* < 0.05; *p < 0.05 significant decrease versus ASI-Control, by post hoc tests.

Considering the available knowledge about pivotal role of glial activation on pathological pain development, in addition of reflexive pain responses, both astrogliosis and microgliosis in the spinal cord were studied in the two models to gain mechanistic insights. Regarding RIM models, spinal cord astrogliosis was evaluated, and significant differences in GFAP immunoreactivity were found at postinduction weeks 4 and 6 (all *p*’s < 0.06; Kruskal–Wallis analysis) (Fig. 2). All RIM models (RIM3, RIM4, RIM6) showed a significant GFAP immunoreactivity increase at 4 weeks when compared with CNT6 (all *p*’s < 0.029; Mann–Whitney U-test) (Fig. 2A), but only RIM6 showed significant astrogliosis at 6 weeks postinduction in comparison with either CNT6 (*p* = 0.01) or RIM3 (*p* = 0.038) (Fig. 2B). In ASI mice, a significant increase in GFAP immunoreactivity was found at 4 weeks postinduction (*p* = 0.043) in comparison to the control (Fig. 2C), and a lack of significance was observed between both groups at 6 weeks postinduction (*p* = 0.394) (Fig. 2D). Regarding spinal cord microgliosis, similar results were obtained. While a transient increase in percentage of reactive microglia was shown after RIM3, RIM4 and ASI models (all *p*’s < 0.021 vs. controls) at 4 weeks post-induction (Fig. 3A), long-lasting (6 weeks) microgliosis was observed only in RIM6 in comparison with either of RIM groups (all *p*’s < 0.006) (Fig. 3B). As for ASI mice, a significant increase in percentage of reactive microglia was observed at 4 weeks postinduction (*p* = 0.029) in comparison to the control (Fig. 3C) but a lack of significance was observed between both groups at 6 weeks postinduction (*p* = 0.200) (Fig. 3D). All these findings reveal that RIM3, RIM4 and ASI models lead to transient spinal cord gliosis up to 4 weeks postinduction, whereas RIM6 pattern administration results in longer-lasting astro- and microgliosis up to 6 weeks.

### Evaluation of long-lasting nonreflexive pain responses in either reserpine-induced myalgia or intramuscular acid saline solution injection mouse models

**Figure 4:**
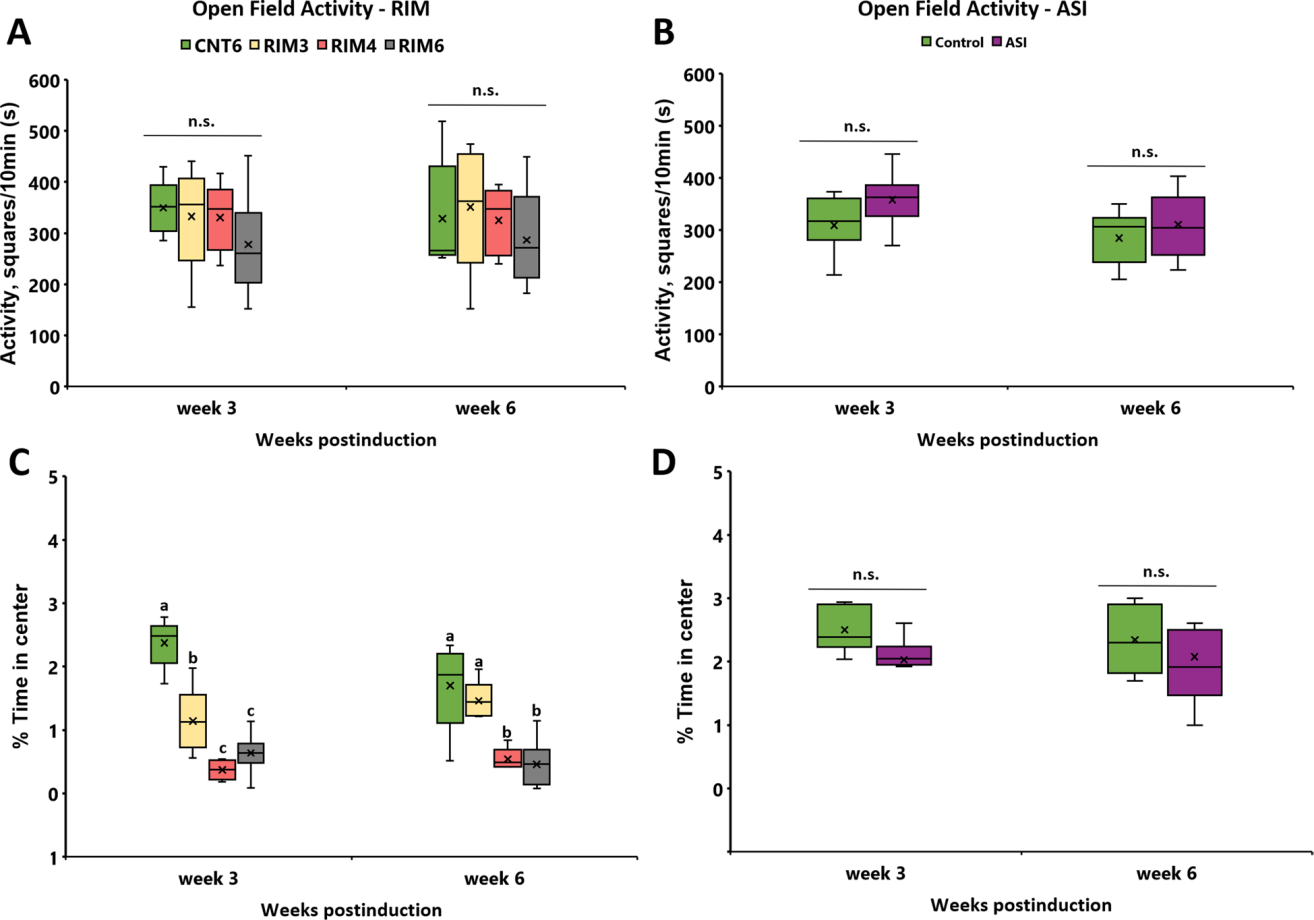
Open field behavioral assessment at 3 and 6 weeks postinduction in mice subjected to reserpine-induced myalgia and intramuscular acid saline solution injection. No locomotor disturbances were found in the (**A**) RIM or (**B**) ASI models. (**C**) RIM3, RIM4 and RIM6 showed significantly decreased time spent in the center when compared with CNT6 at 3 weeks postinduction. At 6 weeks, RIM4 and RIM6 still showed this significant decrease, whereas RIM3 showed no differences in comparison to CNT6. Additionally, the difference between the time spent in zones was significantly increased in the RIM6 group. (**D**) A lack of significance was observed in ASI mice. Data are expressed as the median ± IQR, and the mean is also shown as x. a–c: Groups not sharing a letter showed significant differences in time spent in the OF center, *p* < 0.05.

Since nonreflexive pain responses are usually present in FMS patients, anxiety- and depression-like behaviors were evaluated in RIM and ASI animals. To determine anxiety as a nonreflexive pain response, open field (OF) and dark and light (DL) tests were performed at 3 and 6 weeks after induction-starting to determine either transient or long-lasting behaviors. The number of squares crossed was first evaluated in the OF to determine potential alterations in locomotion that could mask anxious behavior. The Friedman test revealed no significant distribution variation during the experimental period in either RIM (*p* = 0.079) (Fig. 4A) or ASI (*p* = 0.085) (Fig. 4B) models, indicating no locomotor disturbances associated with either of the induction treatments. On the other hand, the time spent in the OF center zone distribution significantly varied during the weeks (*p* < 0.0001; Friedman test) in RIM models, and significant group differences on postinduction weeks 3 and 6 (all *p*’s < 0.002; Kruskal–Wallis test) were found (Fig. 4C). In particular, 3 weeks postinduction, RIM3 (*p* = 0.016), RIM4 (*p* = 0.008) and RIM6 (*p* = 0.001) showed a significant decrease in time spent in the center when compared with CNT6 (Mann–Whitney U-test). Subsequently, while at 6 weeks after induction no difference was found between CNT6 and RIM3 (*p* = 0.310), both RIM4 and RIM6 animal groups still showed a significant decrease in time spent in the center in comparison to controls (all *p*’s < 0.032) (Fig. 4C). In contrast, no significant differences were found in the time spent in the center between the ASI animals and the control group at either 3 or 6 weeks after induction (Fig. 4D). In summary, these findings in the OF test suggest that ASI mice did not develop anxiety-like behaviors, that RIM3 may show transient anxiety up to 3 weeks postinjury and that both RIM4 and RIM6 showed long-lasting anxiety up to 6 weeks after induction-starting.

**Figure 5:**
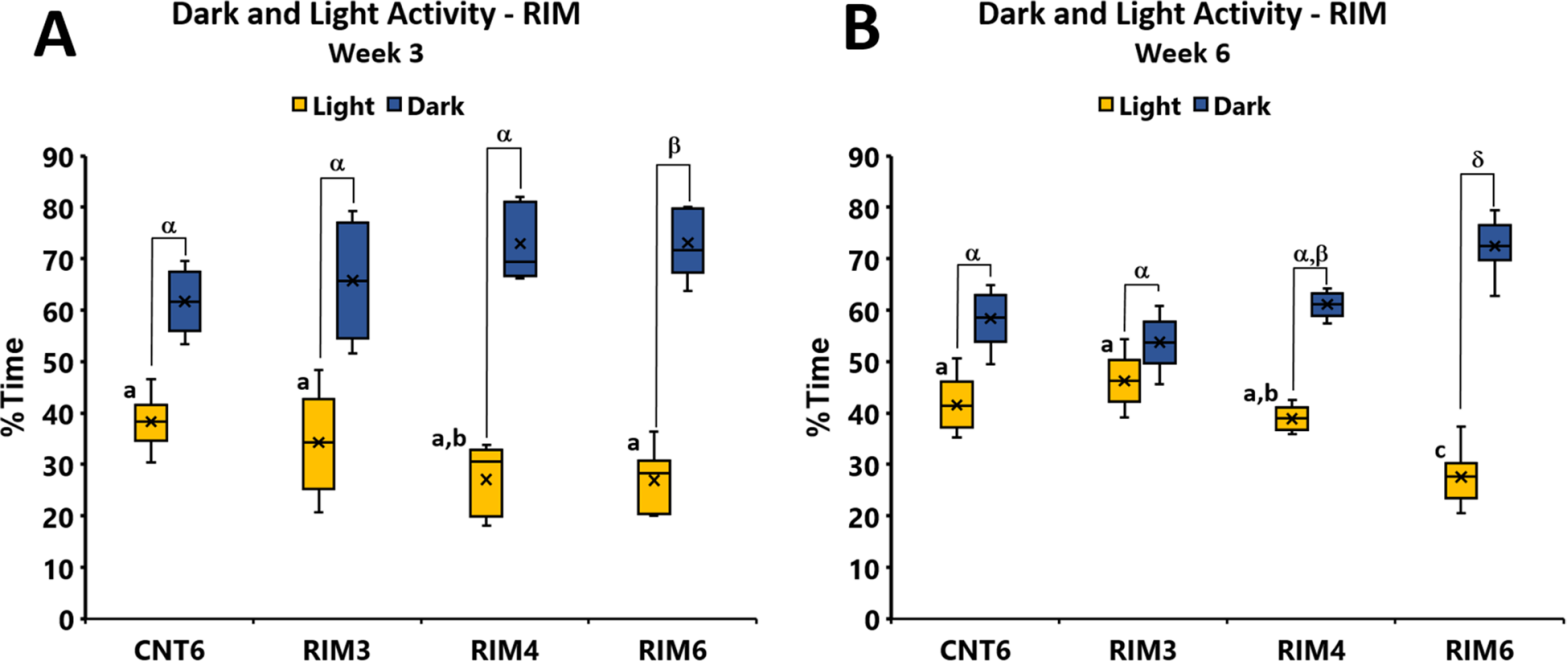
Dark and light behavioral assessment at 3 and 6 weeks postinduction in mice subjected to reserpine-induced myalgia. At (**A**) three and (**B**) six weeks postinduction, RIM6 animals showed a significant decrease in time spent in the light zone compared with CNT6 animals. Additionally, the difference between the time spent in zones was significantly increased in the RIM6 group at both three and six weeks postinduction. Data are expressed as the median ± IQR, and the mean is also shown as a x. a–c: Groups not sharing a letter showed significant differences in time spent in the light zone, *p* < 0.05; α–β: Groups not sharing a letter showed significant differences in time spent in the light zone—time spent in the dark zone *p* < 0.05.

**Figure 6:**
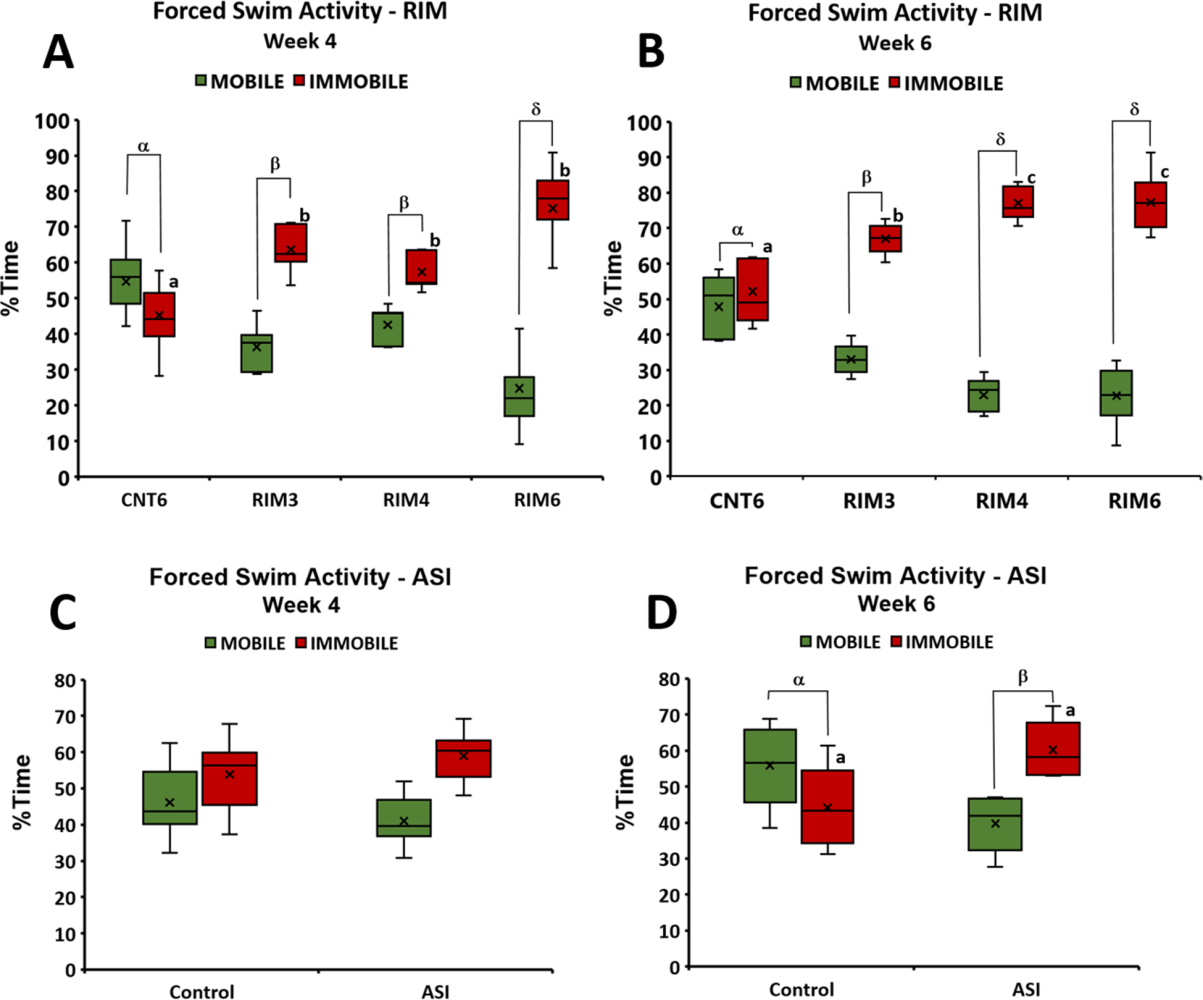
Percentage of immobility and mobility time in the forced swimming test in mice subjected to reserpine-induced myalgia and intramuscular acid saline solution injection. Data are expressed as median ± IQR, and the mean is also shown as a x. a–b: Groups not sharing a letter showed significant differences in %Immobility time, *p* < 0.05; α–β: Groups not sharing a letter showed significant differences in %Immobility-%Mobility time *p* < 0.05.

Regarding the DL test, the time spent in the DL zones distribution significantly varied during the weeks (*p* < 0.014; Friedman test) in the RIM models. Significant group differences were found at postinduction weeks 3 (Fig. 5A) and 6 (Fig. 5B) (all *p*’s < 0.05; Kruskal–Wallis test). RIM6 animals showed a significant decrease in the time spent in the light zone compared with CNT6 animals at both 3 (*p* = 0.012) and 6 (*p* = 0.002) weeks postinduction. The difference in time spent between the dark and light zones at 3 and 6 weeks postinduction was also significantly increased in RIM6 animals compared to the CNT6 (all *p*’s < 0.002) and RIM3 (all *p*’s < 0.001; Mann–Whitney U-test) groups. RIM3 mice did not show significant differences when compared with CNT6 mice at any time point, whereas RIM4 mice were not clearly differentiated from either CNT6 or RIM6 mice. No significant differences in time spent in the dark zone were found between ASI mice and the control group either at 3 or 6 weeks after induction (data not shown). Findings in the DL are similar to those in the OF: ASI and RIM3 mice did not develop anxiety-like behaviors, RIM4 may show transient anxiety up to 3 weeks postinjury, and RIM6 showed long-lasting anxiety up to 6 weeks after induction-starting.

In addition, the forced swimming (FS) test was performed with both RIM and ASI mouse models to determine depression-like behavior, another nonreflexive pain response. Animals were evaluated 4 and 6 weeks after induction-starting to determine either transient or long-lasting depressive-like behavior related to the models’ inductions. Regarding RIM models, group differences were found at postinduction weeks 4 and 6 (all *p* values < 0.001; Kruskal–Wallis test). Specifically, 4 weeks after induction, the percentage of immobility time of all RIM groups (RIM3, RIM4 and RIM6) was significantly increased when compared with CNT6 (all *p*’s < 0.029; Mann–Whitney U-test) (Fig. 6A). The difference between immobility *and* mobility in RIM6 was significantly increased compared with that in any of the other experimental groups. Subsequently, at 6 weeks postinduction, the immobility time of RIM3 was significantly increased compared with that of CNT6 (*p* = 0.007), and both RIM4 and RIM6 showed increased immobility in comparison to that of the RIM3 group (all *p*’s < 0.013) (Fig. 6B). In contrast, while no significant change was observed in ASI 4 weeks after intramuscular acid saline solution injection (*p* = 0.297) (Fig. 6C), a slight increase in depression-like behavior was observed at 6 weeks postinduction in ASI animals (*p* = 0.045) (Fig. 6D). These findings indicate that ASI triggers slight and delayed depressive-like behavior at 6 weeks postinduction. Clear long-lasting depressive-like behavior is shown in mice subjected to reserpine-induced myalgia, with RIM6 showing the most robust and sustained increase in this mood disorder.

### Acute pregabalin administration alleviates thermal hyperalgesia in both the RIM6 and ASI mouse models

Given the results obtained so far and consequently the potential suitability of RIM6 and ASI mice as nocipolastic pain models, a dose–response study was designed to determine whether pathological pain may be modulated by pregabalin (PGB), one of the FDA recommended drugs for fibromyalgia. RIM6 and ASI mice at were treated with PGB (5, 10, 20 and 40 mg/kg) five days after the last administration of the inducer. Then, thermal hyperalgesia was recorded 30 min after intraperitoneal administration of PGB.

**Figure 7:**
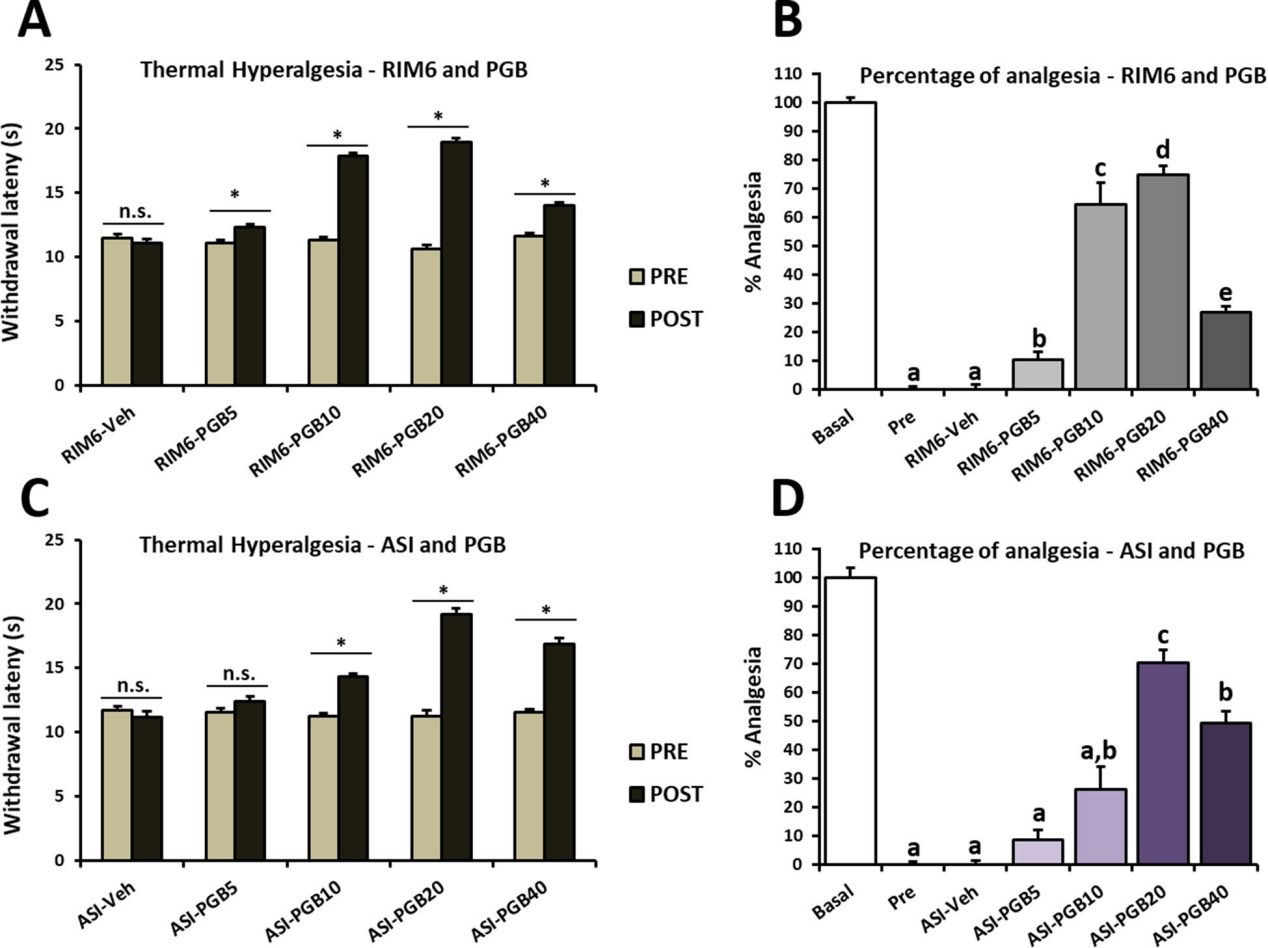
Dose–response effect of pregabalin acute treatment on mice subjected to reserpine-induced myalgia and intramuscular acid saline solution injection. Hind paw withdrawal latency in response to a thermal stimulus pre- and after Pregabalin administration in the **(A)** RIM6 and **(B)** ASI models and **(C,D)** percentage of analgesia 30 min after administration at 28 (RIM6) or 10 (ASI) days postinduction. Data are expressed as the mean ± SEM (n = 5 per group). *p < 0.05 Pre versus Post; n.s. = no significant differences; a–e: Groups not sharing a letter showed significant differences, *p* < 0.05, by post hoc tests.

For RIM6 model mice, while no effects were recorded after vehicle (*p* = 0.715), all PGB doses resulted in significantly increased withdrawal latency to thermal stimulation 30 min after administration (all *p*’s < 0.045) (Fig. 7A). Then, the percentage of antinociceptive effect was calculated over time (time-points basal [i.e. preinjury], pretreatment and 30 min after treatment). The results showed that all doses exerted antinociceptive effects when compared with preadministration and vehicle administration (all *p*’s < 0.001). The highest percentage was shown after 20 mg/kg of PGB, which was also significantly higher than other doses (all *p*’s < 0.032) (Fig. 7B). Similar results were shown in the ASI model. While no effects were recorded after vehicle (*p* = 0.500) or PGB5 (*p* = 0.138), the other doses (PGB10, PGB20 and PGB40) significantly increased withdrawal latency to thermal stimulation 30 min after administration (all *p*’s < 0.05) (Fig. 7C). PGB20 exerted the highest antinociceptive effect in comparison with other doses (all *p*’s < 0.023) (Fig. 7D).

## Discussion

In the context in which the lack of effective treatments to alleviate both reflexive and nonreflexive pain responses associated with fibromyalgia condition is unmistakable, the availability of suitable preclinical models to assess pharmacological strategies is crucial. Although in recent years some remarkable animal models have been developed to mimic the main characteristics of human fibromyalgia^29^ most of them show pain responses in the short term (see Supplementary Table S1). To this end, the present work was aimed to develop two mouse models showing long-lasting reflexive and nonreflexive pain responses after several reserpine (RIM) or intramuscular acid saline solution (ASI) injections. Regarding pain behaviors, our findings reveal that reflexive pain responses (thermal hyperalgesia and mechanical allodynia) in the RIM3, RIM4 and ASI models lasted up to 5 weeks postinduction and up to at least 6 weeks in the RIM6 model. Considering that both pain responses have been induced without primary tissue damage, both RIM and ASI can be suggested as suitable experimental models of fibromyalgia-related nociplastic pain.

Regarding potential mechanisms, it is known that reserpine is a vesicular monoamine reuptake blocker^30^ that binds to the vesicular catecholamine transporter (VMAT2), interfering with the storage of monoamines in intracellular vesicles and causing monoamine depletion in the central nervous system^31^. Hence, reserpine administration likely prevents serotonin and noradrenaline from entering the secretory synaptic vesicles of neurons in the descending inhibitory pain pathway that, from the rostral ventral medulla (RVM) and locus coeruleus (LC), project to nociceptive neurons of the dorsal horn of the spinal cord. The lower the serotonin and noradrenaline release over spinal cord neurons, the lower the descending pain inhibitory effect. That is, in RIM models, reserpine may promote the transmission of painful signals in the spinal cord by decreasing downward inhibition. This hypothesis is supported by the findings reporting depletion of noradrenaline and serotonin in spinal cord of rats after repeated injection of reserpine^15,32,33^. However, in addition to these effects on descending pathways, the potential proalgesic effects of reserpine on the noradrenergic ascending pathways arising from the LC cannot be dismissed. According to available literature, it can be suggested that noradrenaline released by LC neurons on different neurons nuclei (e.g. thalamus, hypothalamus, prefrontal cortex, and anterior cingulate cortex) may facilitate analgesia via corticotropin-releasing factor (CRF) secretion^34^, may stimulate descending oxytocin nerve fibers inhibiting the transmission of pain at the level of the dorsal horn^35^, increase pain perception at the cortical level (PFC, ACC)^36^, and inhibit pain transmission at the thalamic level^37^. Hence, repeated administration of reserpine may affect this ascending noradrenergic pathway resulting in less noradrenaline release on these nuclei, and consequently, may cause a decrease in CRF secretion, less analgesia, decreased stimulation of descending oxytocin neurons of the hypothalamic paraventricular nucleus with increased input of pain signals into the spinal cord, and less thalamic inhibition with increased transmission of pain to the cerebral cortex. Thus, considering that RIM4 and RIM6 animal are variations of the original RIM3 model, the extra and/or booster doses of reserpine received in comparison with RIM3 would cause prolonged depletion of norepinephrine in both ascending and descending noradrenergic pathways. Since RIM4 received lesser number of booster doses than RIM6, RIM4 mice recover norepinephrine levels earlier and consequently, pain responses also normalize earlier compared to RIM6.

For the ASI model, it is known that proton sensing ion channels (e.g., ASIC3 and TRPV1) are involved in activating muscle nociceptors and inducing the central sensitization observed in animal models of chronic muscle pain^38,39^. Repeated intramuscular injection of acidified saline solution may activate ASIC3, leading to a transient inward current followed by a sustained inward current in afferent nociceptive neurons from muscle^40^. While ASIC ion channels are important in the development of hyperalgesia to acid injections, they are not involved in maintaining hyperalgesia to repeated intramuscular acid injections^41^. Other mechanisms, including the upregulation of both Nav1.7 and Nav1.8 ion channels in dorsal root ganglion (DRG) and dorsal horn neurons, may be implicated in the maintenance of hyperalgesia after repeated intramuscular injection of acidified saline solution^40^. Moreover, intramuscular injection of acid saline (pH 4.0) increased the expression of TRPV1 and ASIC3 in DRG neurons, contributing to the generation of hyperalgesia^25,40^. Hence, the repeated injection of acidified saline solution may stimulate the ASIC3 and TRPV1 receptors of muscle nociceptors, causing hyperexcitability of the muscle nociceptive afferent fibers, which is maintained by an overexpression of sodium-dependent voltage channels. All these events may contribute to afferent hyperexcitability in the ASI model, with greater neurotransmitter release in the dorsal horn neurons that may contribute to a greater activation of these neurons, as well as to their central sensitization. Although these peripheral mechanisms can be clearly attributable to the ASI model, it is worth mentioning that it has been described also peripheral mechanisms in reserpine model^17,21,42^. Animals treated with repeated administrations of reserpine may show increased sensitivity of nociceptive afferent fibers to mechanical stimuli mediated by the increase in ASIC3 channels, and also to painful chemical stimuli (formalin). This peripheral hypersensitivity in RIM3 animals may lead to increased hyperactivation and hyperexcitability of dorsal horn neurons (laminae I to VI). Furthermore, this hyperexcitability of spinal neurons also may contribute to the decrease in inhibitory inputs received by these spinal neurons, as well as the reactivation of spinal microglia cells.

In addition to the original alteration resulting in reduced descending inhibition (RIM) or peripheral facilitation (ASI), pathophysiological processes associated with central sensitization develop in both animal models may be important for pathological pain chronicity. Indeed, central sensitization processes may play a pivotal role in FMS and nociplastic pain development^7,12^. In this study, we focused on glial activation^17^ and both astrogliosis and microgliosis in the spinal cord were evaluated in the two models to gain mechanistic insights. Results revealed that that RIM3, RIM4 and ASI models lead to transient spinal cord gliosis up to 4 weeks postinduction, whereas RIM6 pattern administration results in longer-lasting astro- and microgliosis up to 6 weeks. These results are consistent with previous results obtained in the short term in rat models^17,24,43^, but to our knowledge, this is the first study describing spinal cord gliosis up to 6 weeks in an RIM6 mouse model and up to 4 weeks in the ASI. Spinal cord gliosis is known to play a pivotal role in the pathogenesis of chronic pain, and glial activation is now recognized as a powerful mechanism underlying the pathogenesis of chronic pain^44^. Interestingly, there was a coincidence in time (i.e., temporal correspondence) between reflexive pain responses and either astro- or microgliosis in the spinal cord (see Supplementary Fig. S2). Up to 4 weeks postinduction, all experimental groups showing either astro- or microgliosis showed a significant increase in both thermal hyperalgesia and mechanical allodynia. At 6 weeks postinduction, only RIM6 showed significant reflexive pain responses and was the only model showing significant spinal cord gliosis (see Supplementary Fig. S2J,L).

Is there a causal relationship between gliosis and pain? The exact biological mechanisms by which spinal astrocytes and microglia become activated in RIM or ASI mice are unknown. For RIM, decreased release of norepinephrine and serotonin in the dorsal horn of the spinal cord may favor glial cell reactivation^25,45,46^, which synthesizes and secretes inflammatory mediators that favor pain neurotransmission in the dorsal horn and thus the excitability/sensitization of spinal nociceptive neurons^47–50^. It has been suggested that monoaminergic neurons of the RVM release serotonin that interacts with astrocyte 5-HT5A receptors, causing a decrease in cAMP levels in a physiological situation, which keeps them in a nonreactive state^51^. In turn, noradrenaline released by LC neurons may act on the beta-adrenergic receptors of astrocytes, causing an overexpression of the NF-kappa-B inhibitory factor (IκB) that blocks this transcription factor, preventing the production of inflammatory cytokines^52^. At the microglial cell level, noradrenaline may also interact with the beta-adrenergic receptors on these glial cells, causing an inhibition of the phosphorylation of p38-MAPK, a kinase involved in the activation of microglial cells and the generation of inflammatory factors^53^, as well as inhibiting the proliferation of these glial cells^54^. Hence, it is reasonable to hypothesize that the lower release of these biogenic amines in RIM models facilitates the activation of both glial cell types. For the ASI model, glial activation may be explained by the overexcitation of muscle nociceptors, which may release more neurotransmitters in the spinal cord, which in addition to stimulating nociceptive spinal neurons may also stimulate glial cells, as has been suggested in models of inflammatory pain^55–58^. The stimulation of these nociceptors by local acidosis may lead to an elevated release of neurotransmitters in the dorsal horn that interact with glutamate receptors (AMPA and NMDA), purinergic receptors (P2X4 and P2X7) and substance P receptors (NK1) of the microglia cell membrane, causing their activation and the consequent production of cytokines, chemokines, nitric oxide and prostaglandins^59^. These neurotransmitters may also interact with NMDA receptors, purinergic receptors and substance P (NK1) receptors on spinal astrocytes^60^, inducing the activation of these glial cells and subsequent synthesis and release of inflammatory chemical mediators. As mentioned above, most of these released mediators are known to promote dorsal horn pain neurotransmission and sensitization of second-order nociceptive neurons. Accordingly, original alterations resulting in reduced descending inhibition (RIM) or peripheral facilitation (ASI) are crucial for the initiation of pain-related pathophysiological processes associated with central sensitization, but glial activation may exacerbate them and likely contribute to maintaining them over time in the absence of other apparent drivers of chronicity. In addition, it is worth mentioning that not only spinal gliosis may be associated with pain responses development but also plastic changes in supraspinal pain-related areas may be associated^61^. In this context, D'Amico et al^62^ have observed that RIM3 rats show a significant increase in the activation of microglia cells and astrocytes in supraspinal structures, especially in the hypothalamus. Likewise, it has been shown an increase in Iba1-positive cells in brains of these RIM3 rats when compared to control animals, suggesting an increase of cerebral microgliosis^63^.

Although it is clear that the evaluation of reflexive-pain responses is relevant in an FMS-like animal model, so is also the expression of associated mood disorders (i.e., nonreflexive pain responses) observed in patients. The nonreflexive responses are likely to provide different and complementary information about the impact of the sensory abnormality on the whole animal^64^. This emerging consideration has led to increased use of novel approaches to measure the extent to which nociceptive information influences non-evoked behavioral outputs in freely behaving animals^64–69^. Hence, anxiety- and depression-like behaviors were evaluated in both RIM and ASI models since these nonreflexive pain responses or mood disorders are known to be associated with FMS^5,8^. As a result, we observed that RIM4 and especially RIM6, but not ASI, resulted in long-lasting anxiety behavior and that RIM4, RIM6 and ASI develop long-lasting depressive-like behavior, even when reflexive pain responses are not present (RIM4 and ASI). Accordingly, other studies described a different time course of sensory and affective consequences of chronic pain. Affective ones develop later and last longer^70^.

Among the models, RIM6 would be the most suitable fibromyalgia-like model, as it relates to the robustness of long-lasting reflexive and nonreflexive pain responses (see Supplementary Fig. S3). Actually, to our knowledge, RIM6 is the first FMS-like mouse model presenting sensory and affective abnormalities up to 6 weeks. However, it is worth highlighting that RIM4 and ASI models show nonreflexive pain responses at 6 weeks, when reflexive responses are no longer presented. Furthermore, although it has been reported transient locomotor alterations in reserpine injected animals^71,72^, our reported emotional impairments cannot be attributed to reduced locomotor activity since results on 3 and 6 weeks post-induction showed no significant locomotor disturbances. Altogether these results would also support the importance of non-reflexive pain responses in animal models since these symptoms are clinically relevant in chronic pain in humans^4^.

Data obtained so far suggested that both RIM6 and ASI mice could be suitable models for nociplastic pain. To determine whether this pathological pain would be modulated pharmacologically in these models, a new experiment was designed using pregabalin (PGB), one of the FDA recommended drugs for fibromyalgia^13^. To this end, we administered PGB in an acute dose–response study (5, 10, 20 and 40 mg/kg) to both RIM6 and ASI mice at 5 days after the last administration of the inducer. Then, thermal hyperalgesia was recorded 30 min after intraperitoneal administration of PGB. The results indicated that PGB attenuated thermal hyperalgesia dose-dependently in both the RIM6 and ASI models, with PGB20 being the most effective. PGB40 did not provide better benefits than PGB20, likely because it exceeded the selectivity threshold of pregabalin for its primary target, and off-target effects may arise and exert a counteracting/undesired effect. Note that the plasma concentration two hours after i.p. administration of 30 mg/kg to mice is 7.7 μg/mL^73^, which corresponds to 48 μM, and this exceeds the affinity of pregabalin for its primary targets alpha-2-delta-1 and alpha-2-delta-2^74^. Although reflexive pain-response alleviation after pregabalin administration was described in RIM3 rats^15,33,75^, the present study first describes the antihyperalgesic effect of pregabalin in the RIM6 and ASI model in the mouse, thus validating both models as suitable for use in pharmacological studies addressing the alleviation of sensory, nociplastic/FMS pain-related behaviors. Pregabalin binds to alpha-2-delta subunits of the voltage-dependent channels for calcium ions^76^, which causes inhibition of calcium ion entry by these ion channels in presynaptic nerve terminals, which in turn induces a decrease in the release of neurotransmitters such as glutamate or substance P in neurons and glial cells of the dorsal horn^77^. Therefore, pregabalin has been described to reduce not only the excitability and central sensitization of spinal nociceptive neurons^78,79^ but also the glial reactivity mediated by glutamate and ATP. Astrocytes and microglial cells express glutamatergic and purinergic receptors^60,80,81^ and respond to these neurochemical mediators transforming into reactive cells that synthesize and release inflammatory chemical mediators that, in turn, increase the excitability of spinal nociceptive neurons.

In conclusion, to our knowledge this is the first study showing that RIM6 and ASI mouse models show reflexive and nonreflexive responses up to 5–6 weeks, accompanied by either astro- or microgliosis in the spinal cord as pivotal physiopathology processes related to such condition development. In addition, acute treatment with pregabalin resulted in reflexive pain response alleviation in both the RIM6 and ASI models. Consequently, both may be considered suitable experimental models of fibromyalgia-like condition, especially RIM6, and increases the chances of designing new pharmacological strategies against long-lasting fibromyalgia-like symptoms using either RIM or ASI experimental models (see Supplementary Table S2).

## Methods

### Animals

Wild-type (WT) adult female CD1 mice that weighed a median of 22 gr (19–26 gr) and aged 8 weeks were obtained from Janvier Laboratories (France). They were housed in a colony room at 19–22 °C and 40–60% humidity, with a 12:12 h light/dark cycle and ad libitum access to food and water, in groups of five in appropriate plexiglass cages with wood-shaving bedding. Cages were changed twice weekly. All mice were allowed to acclimatize for at least 1 h to the facility rooms before any functional, behavioral, or surgical procedures, which were all conducted during the light cycle. Sentinel mice were routinely tested for pathogens, and facilities remained pathogen free during the whole experimental period. The number of mice used in this study in all procedures was maintained at a minimum, working with experimental groups consisting of 6 to 12 mice. The animal sample size was calculated using GRANMO (Version 7.12 April 2012) and based on the ethical limits exposed by the Animal Ethics Committee.

All experimental procedures and animal husbandry were conducted following the ARRIVE 2.0 guidelines and according to the ethical principles of the IASP. for the evaluation of pain in conscious animals^82^ and the European Parliament and the Council Directive of 22 September 2010 (2010/63/EU) and were approved by the Animal Ethics Committee from the University of Barcelona (CEEA: 35/16; DAMM: 8887). All efforts were made to minimize animal suffering and to keep the number of animals to a minimum to demonstrate consistent effects for the procedures and treatments.

### Induction of fibromyalgia-like condition (FM and ASI) and pharmacological treatment

Reserpine was purchased from Sigma-Aldrich (St. Louis, MO, USA), dissolved in acetic acid, diluted to a final concentration of 0.5% acetic acid with saline solution, and injected subcutaneously at 0.25 mg/kg^20^. Animals received repeated subcutaneous injections of reserpine^20^ three times (on days 0 [on the day of the first injection], 1, 2; group RIM3), four times (on days 0, 1, 2 and 9; group RIM4) and six times (on days 0, 1, 2, 9, 16 and 23; group RIM6). Control animals received reserpine dilution vehicle (group CNT6), employing the same schedule of administration as group RIM6. On the other hand, other groups of animals were injected with acid saline solution (pH = 4, 10 µL; group ASI) into the right gastrocnemius muscle using a Hamilton syringe on days 0 and 5^83^. Control animals received saline solution (CNT-ASI group).

For pharmacological treatment, five days after the last injection of reserpine or acidified saline solution, a set of different mice were treated with pregabalin (i.p.) at doses of 5, 10, 20 and 40 mg/kg, whereas control mice received a vehicle dilution of pregabalin (saline solution). Thirteen minutes after administration, thermal hyperalgesia was evaluated as described below.

### Evaluation of reflexive pain response: mechanical allodynia and thermal hyperalgesia

#### Thermal hyperalgesia

Thermal hyperalgesia was assessed by determination of hind paw withdrawal latency in response to a thermal stimulus (radiant heat) administered via a plantar test analgesia meter (#37,370; Ugo Basile, Comerio, Italy) according to the Hargreaves method^84–86^. Mice were placed into test enclosures, with the temperature-controlled (29 °C) glass surface of the plantar test device positioned directly underneath. The animals were then allowed to acclimatize for 45 min. The radiant heat source was then positioned under the plantar surface of the animal’s hind paw and activated. A light beam intensity that elicited baseline paw withdrawal latencies of 14–15 s was used. A maximum limit of 20 s was imposed to prevent tissue damage in the absence of a withdrawal response. Both paws were evaluated, and the sum of the mean withdrawal latencies for both hindpaws was determined from the average of three separate trials conducted at 5-min intervals.

#### Mechanical allodynia

Mechanical allodynia was assessed via hind paw withdrawal from von Frey filament stimulation^85–87^. Mice were placed in test chambers with a metal mesh floor through which von Frey monofilaments (bending force range 0.04–2 g) were applied to the plantar surface. Paw withdrawal thresholds were measured using the up-down method paradigm in which 0.4 g was applied first. The response to this filament determined which filament was applied next; a weaker filament was applied if the animal had responded to the previous filament, and a stronger filament was applied if the animal did not respond to the previous filament. Clear paw withdrawal, shaking or licking were considered to be a response. This up-down procedure was limited to four assessments after the first response. Each filament was applied for 2 s, with interstimulus intervals of 5–10 s. Both hind paws were tested. The 50% paw withdrawal threshold was calculated using the Dixon formula: 50% paw withdrawal threshold (g) = [(10^(Xf + κδ)^/ 10 000)], where Xf is the value (in logarithmic units) of the final von Frey filament used, κ is a fixed tabular value for the pattern of positive/negative responses and δ is the mean difference (in log units) between stimuli.

#### Nonreflexive pain responses evaluation

The anxiety response was evaluated by means of the open field and dark/light box tests, whereas depression was evaluated by the forced swimming test. For the *open field test*, the mice were placed in the corner of a white quadratic box (50 × 50 × 45 cm) in which its arena had been previously divided into 5 × 5-cm squares by black lines. Mice were then allowed to move freely for 5 min under dim and dispersed light conditions (room light 45–50 lx), while the number of square crossings was counted by an observer in a blinded manner. The behavior during the whole session was recorded with a video camera and then analyzed. The time spent in the wall and the center (10 × 10-cm virtual square in the middle of the open field) regions and the latency to enter the center zone were interpreted as anxiety-like behaviors. In addition, the number of square crossings was used as an index of locomotion activity. After each trial (10 min of length), the open field was cleaned with 5% ethanol/water. The open field exploration test provides the ability to systematically assess novel environment explorations and general locomotion activity, and it also provides an initial screen for anxiety-like behavior in rodents^88^. In the *dark/light box test*, a 27 × 27 × 26-cm lit (room light 45–50 lx) white compartment with an open top was connected through an opening entrance (5 × 5 cm) to a 27 × 27 × 26-cm black box compartment covered with a lid. The mice were placed in the center of the dark compartment and were therefore allowed to freely explore the apparatus for 10 min. The behavior during the whole session was recorded with a video camera and subsequently analyzed. The time spent in the light compartment and the latency to enter the light compartment were both considered indicators of anxiety-like behavior. After each 10-min trial, both compartments were cleaned with 5% ethanol/water^88^. Finally, in the *forced swimming test*, mice were individually forced to swim in open cylinders (40 cm height × 15 cm diameter) containing 30 cm of water at 25 ± 1 °C. Their behavior during the whole 6-min test was recorded with a video camera (Sony HDR-CX190) and then analyzed. The immobility time was determined whenever no additional activity other than the necessary movements to keep the mouse’s head above the water was observed. The forced swimming test is a useful procedure for evaluating depression-like behaviors^89,90^. Behavioral tests were performed before the induction of fibromyalgia, at 3 and 6 weeks postinduction of fibromyalgia for the open field and dark/light box tests, and at 6 weeks postinduction of fibromyalgia for the forced swimming test.

#### Histological evaluation

At the end of functional and behavioral evaluation, animals were deeply anesthetized with pentobarbital (90 mg/kg; i.p.), the spinal cord was quickly removed, and the segment distal to T10 was fixed in Zamboni solution for histological evaluation. After fixation in Zamboni solution^91^ for 14 days, distal spinal cord segments were bathed in a cryoprotective solution of 30% sucrose in phosphate buffered saline (PBS, 0.1 M, pH = 7.4) for another 14 days and then cut in a cryostat (CM1520, Leica, Barcelona). Histological sections (60 µm) were processed by immunohistochemical techniques to observe astrocytes (glial fibrillary acidic protein or GFAP; 1:200, AB7260, ABCAM) and microglial cells (Iba-1; 1:200, Cat# 019–19,741; WAKO, Richmond, VA, USA). As a specificity control, some spinal cord sections were incubated without primary antibody. Samples were viewed under a microscope equipped with epifluorescence using appropriate filters (Leica DMR-XA; Leica Microsystems, Barcelona). Using a digital camera coupled to the microscope (FMVU-13S2C-CS, Point Gray Research, Canada), images (× 200) were captured from dorsal and ventral horns of GFAP- and Iba-1-immunostained histological sections. A minimum of five histological sections from each animal were evaluated. Images were analyzed using NIH Image software (ImageJ; version 1.37; National Institutes of Health, USA) to determine the area of GFAP and Iba-1 immunoreactivity as described previously^92^. In addition, from Iba-1 immunostained histological sections, the percentage of reactive and nonreactive microglial cells was also determined. Nonreactive microglia cells show a ramified morphology, whereas reactive microglia show shorter thicker processes and an amoeboid shape^60,93^.

### Statistical analysis

All functional and histological analyses were performed in a blinded manner using a numeric code for each mouse. The normal distribution of the data was analyzed by the Kolmogorov–Smirnov test before further applying parametric or nonparametric statistical analyses. When data followed a normal distribution, repeated measures MANOVA (Wilks’ criterion) and analysis of variance (ANOVA) followed by Duncan’s test when applicable were used for data analysis. On the other hand, when data did not follow a normal distribution, they were analyzed by means of the Friedman statistic test for nonparametric repeated measures and Kruskal–Wallis test followed by the Mann–Whitney U test when applicable. In the pharmacological study, the percentage of antihyperalgesic effect exerted by a treatment was calculated as follows: % effect = [(PWD − PWV)/(PWN − PWV)] × 100, where PWD and PWV are the paw withdrawal latency (s) in drug-treated and pretreated animals, respectively, and PWN is the paw withdrawal in naïve animals. Pre-/postpharmacological analyses were performed by the Wilcoxon test. In all analyses, the significance level α was set at 0.05, and the statistical program used was SPSS 25.0 for Windows.

## Supplementary Information

Supplementary Information.

## Data Availability

All data generated or analyzed during this study are included in this published article.
