# Supplementary material for: Long-lasting reflexive and nonreflexive pain responses in two mouse models of fibromyalgia-like condition
Source: Sci Rep. 2022 Jun 12;12:9719. doi: 10.1038/s41598-022-13968-7 (PMC9189106; doi:10.1038/s41598-022-13968-7)
Supplement: Supplementary file 1 — Supplementary Information. [file 41598_2022_13968_MOESM1_ESM.pdf]

Supplementary Material

Long-lasting reflexive and nonreflexive pain responses in two mouse models of fibromyalgia-like condition

Beltrán Álvarez-Pérez[1,ǂ], Meritxell Deulofeu[1,ǂ], Judit Homs[1,2], Manuel Merlos[3], José Miguel Vela[3], Enrique Verdú[1,*], Pere Boadas-Vaello[1*]

[1]Research Group of Clinical Anatomy, Embryology and Neuroscience (NEOMA), Department of Medical Sciences, Universitat de Girona, Girona, Catalonia, Spain.

[2]University School of Health and Sport (EUSES), University of Girona, Girona, Catalonia, Spain

[3]WeLab Barcelona, Parc Científic de Barcelona, Catalonia, Spain.

[ǂ]These authors contributed equally to this work

[*]**Corresponding authors:** Dr. Pere Boadas-Vaello and Dr. Enrique Verdú. Grup de Recerca d'Anatomia Clínica, Embriologia i Neurociència (NEOMA), Departament de Ciències Mèdiques, Facultat de Medicina, Universitat de Girona (UdG), Emili Grahit 77, 17003 Girona, Catalunya, Spain. Tel. : +34 972 41 96 22. Lab: +34 972 41 9562. E-mail address: pere.boadas@udg.edu; enric.verdu@udg.edu

SUPPLEMENTARY FIGURES

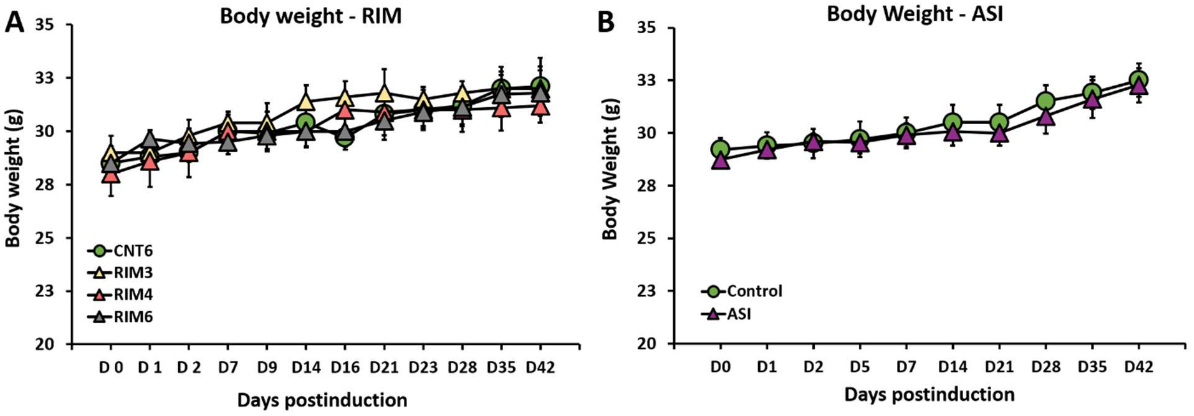

Supplementary Figure S1. Time course of animals weigh of (**A**) RIM models and (**B**) ASI model. No significant effects on body weight related to reserpine or acidified solution administration were detected neither in RIM nor ASI models during the respective experimental period.

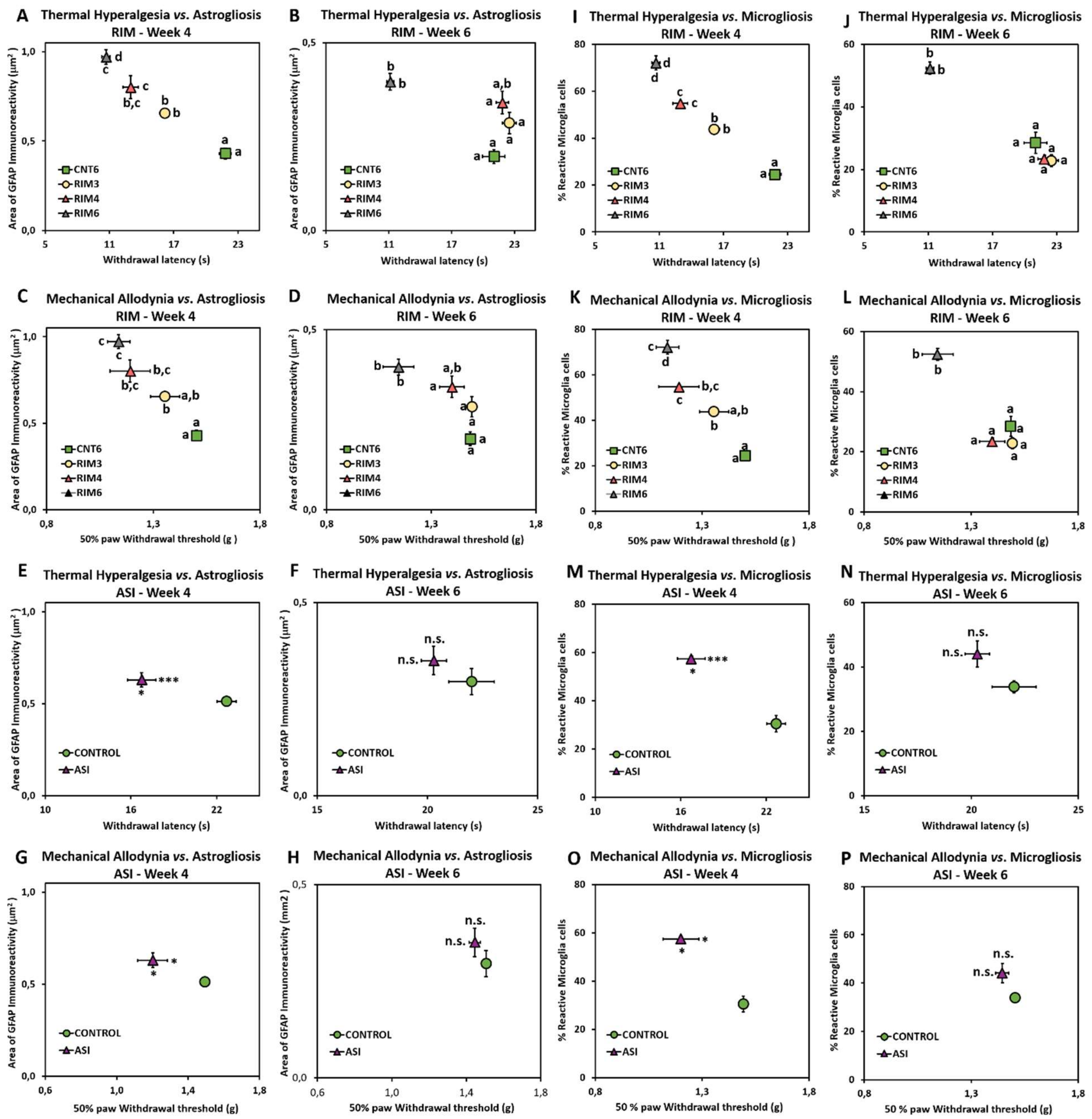

Supplementary Figure S2. Relationship between reflexive-pain responses and spinal cord gliosis in RIM and ASI experimental models 4 and 6 weeks after induction. (**A-H**) Relationship between reflexive pain responses and spinal cord astrogliosis in RIM and ASI models. (**I-P**) Relationship between reflexive pain responses and the percentage of reactive microglial cells in the spinal cord of the RIM and ASI models. Letters or asterisks above the symbols correspond to gliosis, and letters or asterisks to the right of the symbol refer to reflexive pain response scores. Data are expressed as the mean ± SEM. a–d: Groups not sharing a letter are significantly different, $p<0.05$, according to post hoc test; *** $p<0.0001$, * $p<0.05$ significant decrease *vs*. ASI-Control, by post hoc tests.

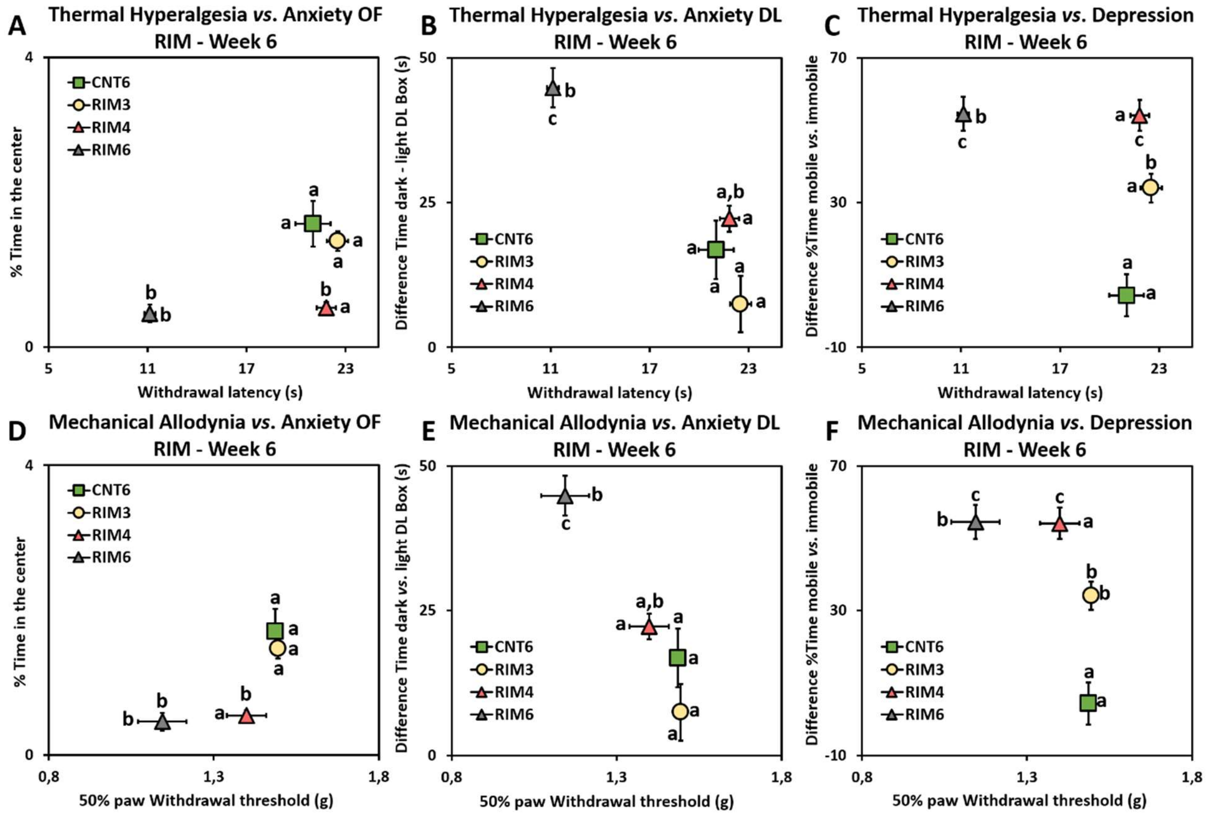

Supplementary Figure S3. Relationship between reflexive and nonreflexive pain responses in RIM experimental models 6 weeks after induction. Relationship between thermal hyperalgesia and (**A**) time in the OF center, (**B**) difference in dark/light DL box compartments, and (**C**) difference in time mobility in the forced swim test in RIM animals. Relationship between mechanical allodynia and (**D**) time in the OF center, (**E**) difference dark/light DLbox compartments, (**F**) difference time mobility in the forced swim test in RIM animals. Letters above the symbols correspond to nonreflexise outcomes, and letters to the right of the symbol refer to reflexive pain response scores. Data are expressed as the mean ± SEM. a–c: Groups not sharing a letter are significantly different, $p<0.05$, according to post hoc test; *** $p<0.0001$, * $p<0.05$ significant decrease *vs*. ASI-Control, by post hoc tests.

SUPPLEMENTARY TABLES

Supplementary Table S1. Overview of current Fibromyalgia-like model (FM-like models)

FM-like model	Induction	Protocol	Experimental period	Reflexive pain responses	Nonreflexive pain responses	Histological/Molecular results	Ref.
**Reserpine induced myalgia (RIM) rat**	Reserpine s.c. 1 mg/kg)	Injection of reserpine for 3, 4 or 6 consecutive days	7-28 days	Thermal hyperalgesia, mechanical allodynia, muscle hyperalgesia	Depression-like behavior	Reduction of biogenic amines in CNS	Nagakira et al 2009, 2012, 2019
**Reserpine induced myalgia (RIM) mouse**	Reserpine s.c. 0.25 mg/kg		4-42 days	Thermal hyperalgesia, mechanical allodynia	Depression-like behavior	Reduction of biogenic amines in CNS	de Souza et al 2014, Nagakura et al 2018
**Acidic Saline Intramuscular injection (ASI) rat**	Acidic saline i.m.	Two acid saline (pH: 4) injections in gastrocnemius muscle (0, 5 days)	6-45 days	Mechanical allodynia, muscle hyperalgesia	Anxiety- and depression-like behavior	No changes in microglia and astrocyte reactivation in spinal cord in acidic saline and control animals	Sluka et al 2001, Nielsen et al 2004, Ledeboer et al 2006, Jasper and MacNeil 201210/5/2022 Liu et al 2014; Murasawa et al 2021
**Acidic Saline Intramuscular injection (ASI) mouse**	Acidic saline i.m.		8-42 days	Mechanical allodynia, muscle hyperalgesia	Not evaluated	Increased c-FOS expression in dorsal horn ipsilateral to muscle injection	Sluka et al 2003; Sharma et al 2009; Chen et al 2010
**Intermittent cold stress (ICS) mouse**	Thermal stress (cold 4ºC and 24ºC)	The animals remain in a cold room (4ºC) overnight (from 4:30pm to 10:00 am), followed by environmental temperatures (22 - 24 °C), alternating between both temperatures every 30 minutes from 10:00 am to 4:30 pm. These procedures are repeated three times. On day four, the animals are adapted to environmental temperature for one hour before behavioural tests.	5-12 days	Thermal hyperalgesia, mechanical allodynia	Not evaluated	Increased TRPV1 expression in profrontal cortex, somatosensory cortex, hippocampus, thalamus. Reduced cross-sectional area of skeletal muscle. Increased corticosterone levels. Reduction of BDNF and CREB expression in hippocampus.	Nishiyori and Ueda 2008; Nishiyori et al 2010, 2011; Montserrat-de la Paz et al 2015; Mukae et al 2015; Bonaterra et al 2016; Lee et al 2017; Lottering and Lin 2021; Hsiao and Lin 2022
**Intermittent cold stress (ICS) rat**	Thermal stress (cold 4ºC and 24ºC)		6-14 days	Increased mechanical sensitivity of type C nociceptors.	Increased depression-like behavior	No changes in BDNF expression in brain areas	Nasu et al 2019; Wakatsuki et al 2021
**Constant cold stress (CCS) mouse**	Thermal stress (cold 4ºC)	The animals remain in a cold room (4ºC) without alternating the environmental temperature for three consecutive nights.	12 days	Transient allodynia	Not evaluated	Not evaluated	Nishiyori and Ueda 2008; Nishiyori et al 2011
**Sound stress rat**	Pure tones at freq. 5-19 Hz and amplitudes 20-110 dB	Exposure to sound stress occurs over four days, on days one, three, and four. The animals are placed in a cage 25 cm from a speaker that emits pure tones at frequencies of 5, 11, 15, and 19 kHz. The amplitudes used varied through time from 20 up to 110 dB at random times each minute that last five or ten seconds. The total time of exposure usually used is 30minutes per day	14 days	Mechanical allodynia and muscle hyperalgesia; increased visceromotor response	Increased anxiety-like behavior	Noradrenaline enhances skin and muscle hyperalgesia	Khasar et al 2005, 2008, 2009; Green et al 2011
**Sound stress mouse**	Pure tones at freq. 5-19 Hz and amplitudes 20-110 dB		29-50 days	Thermal hyperalgesia, mechanical allodynia	Increased anxiety-like behavior	Not evaluated	Hung et al 2020

**Swim stress rat**	Forced swim	Animal is subjected to a forced swim procedure for 10 min by placing them in a plastic cylinder (diameter 30 cm, height 50 cm) that contained 20 cm of water at 24 ±26°C. This procedure is repeated on days 2 and 3. On day 1 it is 10 minutes of swimming, and on days 2 and 3 20 minutes of swimming.	1-27 days	Thermal and chemical hyperalgesia; Mechanical hyperalgesia; Visceral hypersensitivity; incresed pain responses in formalin test	Not evaluated	Higher corticosterone level; increased c-FOS immunoreactivity in dorsal horn	Quintero et al 2000, 2003, 2011; Suarez-Roca et al 2006; Guevara et al 2015; Ji et al 2018; Xu et al 2020
**Hyperalgesic priming rat**	Long-lasting hypersensitivity to proinflammatory mediators (e.g. carrageenan, IL-6, GDNF, and monocyte chemoattractant protein 1)	Inflammatory mediators are administered by intramuscular or intradermal routes and produce transient mechanical hyperalgesia that lasts three-five days. After the hyperalgesia resolves, the inflammatory mediator PGE2 is injected at the same site and produces a markedly prolonged hyperalgesic response that lasts at least one week	1h-20 days	Transient hyperalgesia up to 24h by carrageenan injection in dorsum of hindpaw. PGE2 administration in hindpaw enhances hyperalgesia. Increased hyperalgesia up to 5 days post-injection of carrageenan. PGE2 Injection at 15 days causes hyperalgesia. Intramuscular injection of IL6 or carrageenan causes transient hyperalgesia up to 5 days. Mechanical muscle hyperalgesia after injection of ryanodine in dorsum of hindpaw. PGE2 injection 4-5 days later also increases mechanical hyperalgesia. Gastrocnemius injection of MCP1 causes transient muscle hyperalgesia.	Not evaluated	Not evaluated	Aley et al 2000; Parada et al 2003; Dina et al 2008; Alvarez et al 2014; Ferrari et al 2016;
**Hyperalgesic priming mouse**	Long-lasting hypersensitivity to proinflammatory mediators (e.g. carrageenan, IL-6, GDNF, and monocyte chemoattractant protein 1)		1h-14 days	PAR2 agonist injection in hindpaw causes a transient hyperalgesia and PGE2 injection induces mechanical hyperalgesia up to 14 days. Carrageenan injection triggered mechanical hyperalgesia for 72h. At 6 days, the PGE2 injection causes hyperalgesia that lasts until day 7. IL6r injection causes transient hyperalgesia up to 3 days. Administration of PGE2 7 days after IL6r enhance hyperalgesia. Placlitaxel administration causes hyperalgesia up to 28 days. When at 28 days PGE2 was injected also increases hyperalgesia up to 1 day more.	Not evaluated	After hyperalgesic priming by IL6r, the injection of PGE2 increase microglia reactivity in spinal cord.	Tillu et al 2015; Sikandar et al 2018; Paige et al 2018; Inyanga et al 2019
**Chronic restraint stress rat**	Immobilization in Plexiglas tubes	Animals are restrained by one hour daily over five weeks into Plexiglas tubes	7-35 days	Increased mechanical allodynia but not muscle hyperalgesia. Enhancement of hyperalgesia to formalin test. Increased thermal and mechanical hyperalgesia.	Increased aggressive behavior	Reduction of GFAP-ir in PAG and RVM	Gameiro et al 2005; Bardin et al 2009; Imbe et al 2012, 2013; Huang et al 2021
**Chronic restraint stress mouse**	Immobilization in Plexiglas tubes		10-42 days	Increased mechanical hyperalgesia. Increased thermal hyperalgesia. Higher activity of GABAergic neurons in amygdala	Increased depression-like behavior; Increased anxiety-like behavior	Degeneration and necrosis of neurons in amygdala. Reduction of glutamatergic transmission in PAG	Carneiro de Oliveira et al 2017; Huang et al 2019; Liu et al 2022; Peng et al 2022
**Continuous stress-loading rat**	Swim with a weight on the tail	The animals are kept in cages filled with warm water. The animals swim with a load of steel rings weighing about 8% of their body weight. These rings are attached to their tails.	5-14 days	Increased fatigue over time. Increased mechanical allodynia up to 10 days. Increased muscle hyperalgesia.	Not evaluated	Hyperactivation of proprioceptors in the DRG. Activation and accumulation of microglial cells in dorsal horn.	Tanaka et al 2003; Yasui et al 2014, 2019
**Intermittent psychological stress mouse**	Stressed animals visualizing other animals receiving physical stress and feeling pain	Mice are exposed to intermittent psychological stress once a day for five days using a communication box with nine compartments divided by transparent walls. Mice recipients from psychological stress are put in 4 compartments with a plastic cover to see, hear, and smell the other five mice receiving physical stress (electric footshock).	19 days	Thermal hyperalgesia up to 19 days. Mechanical allodynia and muscle hyperalgesia up to 7 days	Not evaluated	Not evaluated	Ueda and Neyama 2017

Supplementary Table S2. Summary of RIM and ASI available models (including the present study)

FM-like model	Induction by	Protocol	Experimental period	Reflexive pain responses	Nonreflexive pain responses	Histological/Molecular results	Ref.
RIM3 rat	Reserpine s.c.	0.1, 0.3, 1 mg/kg in acetic acid four consecutive days (0, 1, 2, 9 days)	21 days	Tactil allodynia < 7 days at 0.1 and 0.3 mg/kg; and up to 14-21 days at 1 mg/kg	Increased depressive like-behavior	Reduction of biogenic amines in CNS of reserpinized rats	Nagakura et al 2009
RIM3 rat	Reserpine s.c.	1 mg/kg in acetic acid three consecutive days (0,1, 2 days)	28 days	Tactil allodynia up to 28 days, mucle hyperalgesia up to 28 days, and cold hyperalgesia up to 28 days.	Not evaluated	Not evaluated	Nagakura et al 2012
RIM3 rat	Reserpine s.c.	0.5, 1 and 2 mg/kg in acetic acid three consecutive days (0, 1, 2 days)	10 days	Thermal hyperalsia, mechanical allodynia and muscle hyperalgesia up to 10 days	Increased depressive like-behavior	Increased SP levels in hippocampus; decreased levels of noradrenaline, serotonin and dopamine in cortex.	Arora and Chopra 2013
RIM3 rat	Reserpine s.c.	1 mg/kg in acetic acid three consecutive days (0,1, 2 days)	14 days	Tactil allodynia up to 14 days	Not evaluated	Not evaluated	Kaneko et al 2014
RIM3 rat	Reserpine s.c.	1 mg/kg in acetic acid three consecutive days (0,1, 2 days)	7 days	Tactil allodynia and muscle hyperalgesia up yo 7 days	Increased depressive like-behavior	Not evaluated	Blasco-Serra et al 2015
RIM3 rat	Reserpine s.c.	1 mg/kg in acetic acid three consecutive days (0,1, 2 days)	21 days	Tactil allodynia, muscle hyperalgesia, and thermal hyperalgesia up to 21 days	Not evaluated	Enhancement of gliosis in spinal cord. Up-regulation of GABA-A receptors and down-regulation of KCC2 expresion in spinal cord. Reduction of NE, dopamine and serotonin brain and spinal cord	Oe et al 2010; Hernandez-Leon et al 2018; De La Luz-Cuellar et al 2019
RIM3 rat	Reserpine s.c.	1 mg/kg in acetic acid three consecutive days (0,1, 2 days)	17 days	Allodynia up to 17 days	Elevation of rat grimace scale (RGS)	Not evaluated	Nagakura et al 2019
RIM4 rat	Reserpine s.c.	1 mg/kg in acetic acid four consecutive days (0, 1, 2, 9 days)	28 days	Tactil allodynia up to 28 days, mucle hyperalgesia up to 28 days, and cold hyperalgesia up to 28 days.	Not evaluated	Not evaluated	Nagakura et al 2012
RIM6 rat	Reserpine s.c.	1 mg/kg in acetic acid six consecutive days (0, 1, 2, 9, 16, 23 days)	28 days	Tactil allodynia up to 28 days, mucle hyperalgesia up to 28 days, and cold hyperalgesia up to 28 days.	Not evaluated	Not evaluated	Nagakura et al 2012
RIM3 mouse	Reserpine s.c.	0.25 mg/kg in acetic acid three consecutive days (0, 1, 2 days)	4 days	Mechanical allodynia and thermal hyperalgesia up to 4 days	Depression-like up to 4 days	Reduction of dopamine and serotonine contents in brain	De Souza et al 2014
RIM3 mouse	Reserpine s.c.	0.1, 0.5 and 1 mg/kg in acetic acid three consecutive days (0, 1, 2 days)	60 days	Not evaluated	Reduction of locomotion activity at 2 and 20 days	Reduction of TH at 2 and 20 days	de Freitas et al 2016
RIM3 mouse	Reserpine s.c.	0.25 mg/kg or 0.125 mg/kg in acetic acid three consecutive days (0, 1, 2 days)	9 days	Mechanical allodynia at 0.25 mg/kg but not at 0.125 mg/kg at 5 and 7 days post-injection	Not evaluated	Reduction of noradrenaline in spinal cord	Nagakura et al 2018
RIM3 mouse	Reserpine s.c.	0.25 mg/kg in acetic acid three consecutive days (0, 1, 2 days)	42 days	Thermal hyperlagesia and mechanical allodynia up to 28 days	Transient anxiety-like behavior	Spinal cord gliosis	Present study (Álvarez-Pérez et al 2022)
RIM4 mouse	Reserpine s.c.	0.25 mg/kg in acetic acid four consecutive days (0, 1, 2, 9 days)	42 days	Thermal hyperlagesia and mechanical allodynia up to 35 days	Long-lasting anxiety-like behavior, and depressive-like behavior	Spinal cord gliosis	Present study (Álvarez-Pérez et al 2022)
RIM6 mouse	Reserpine s.c.	0.25 mg/kg in acetic acid six consecutive days (0, 1, 2, 9, 16, 23 days)	42 days	Thermal hyperalgesia and mechanical allodynia beyond 42 days	Long-lasting anxiety-like behavior, and depressive-like behavior	Spinal cord gliosis	Present study (Álvarez-Pérez et al 2022)
ASI rat	Acidic saline i.m.	Two acid saline (pH: 4) injections in gastrocnemius muscle (0, 5 days)	42 days	Mechanical hyperalgesia at firts and second injection of acidic saline solution that persist up to 4 weeks after de second injection	Normal locomotion in both experimental groups	Mild tissue injury ranging from single necrotic muscle fibers, to small foci of necrotic fibers, to lymphocytic perimysial inflammation from ipsi. muscle	Sluka et al 2001
ASI rat	Acidic saline i.m.	Two acid saline (pH: 4) injections in gastrocnemius muscle (0, 5 days)	35 days	Mechanical allodynia and muscle hyperalgesia up to 35 days	Not evaluated	Not evaluated	Nielsen et al 2004
ASI rat	Acidic saline i.m.	Two acid saline (pH: 4) injections in gastrocnemius muscle (0, 5 days)	45 days	Mechanical allodynia up to 45 days	Not evaluated	No changes in microglia and astrocyte reactivation in spinal cord in ac. saline and control rats	Ledeboer et al 2006
ASI rat	Acidic saline i.m.	Two acid saline (pH: 4) injections in gastrocnemius muscle (0, 5 days)	6 days	Muscle hyperalgesia at 0 and 5 days post-injection of acid saline solution	Not evaluated	Not evaluated	Jasper and MacNeil 2012
ASI rat	Acidic saline i.m.	Two acid saline (pH: 4) injections in gastrocnemius muscle (1, 6 days)	34 days	Mechanical allodynia up to 34 days	Increased anxiety-like behavior, anhedonic-like behavior and depression-like behavior	Not evaluated	Liu et al 2014
ASI rat	Acidic saline i.m.	Two acid saline (pH: 4) injections in gastrocnemius muscle (0, 5 days)	7 days	Mechanicall allodynia at 7 days	Increased anxiety-, depression- and cognitive-like behavior	Not evaluated	Murasawa et al 2021
ASI mouse	Acidic saline i.m.	Two acid saline (pH: 4) injections in gastrocnemius muscle (0, 5 days)	12 days	Mechanical hyperalgesia	Not evaluated	Primary afferent fibers innervating muscle express ASIC3 and respond to low pH with ASIC-like currents	Sluka et al 2003
ASI mouse	Acidic saline i.m.	Two acid saline (pH: 4) injections in gastrocnemius muscle (0, 5 days)	15 days	Mechanical allodynia and muscle hyperalgesia up to 15 days	Not evaluated	Acidic saline injection induces c-Fos expression in spinal cord ipsilateral to mucle injection	Sharma et al 2009
ASI mouse	Acidic saline i.m.	Two acid saline (pH: 4) injections in gastrocnemius muscle (0, 5 days)	8 days	Muscle hyperalgesia at 0 and 5 days post-injection of acid saline solution	Not evaluated	Increased c-FOS expression in dorsal horn ipsilateral to muscle injection	Chen et al 2010
ASI mouse	Acidic saline i.m.	Two acid saline (pH: 4) injections in gastrocnemius muscle (0, 5 days)	42 days	Thermal hyperalgesia and mechanical allodynia up to 35 days	Depressive-like behavior	Spinal cord gliosis	Present study (Álvarez-Pérez et al 2022)

Tables references

Aley, K.O., Messing, R.O., Mochly-Rosen, D. & Levine, J.D. Chronic hypersensitivity for inflammatory nociceptor sensitization mediated by the epsilon isozyme of protein kinase C. *J. Neurosci*. **20**, 4680-5 (2000).

Alvarez, P., Green, P.G. & Levine, J.D. Role for monocyte chemoattractant protein-1 in the induction of chronic muscle pain in the rat. *Pain*. **155**, 1161-1167 (2014).

Arora, V. & Chopra, K. Possible involvement of oxido-nitrosative stress induced neuro-inflammatory cascade and monoaminergic pathway: underpinning the correlation between nociceptive and depressive behaviour in a rodent model. *J. Affect Disord*. **151**, 1041-52 (2013).

Bardin, L., Malfetes, N., Newman-Tancredi, A. & Depoortère, R. Chronic restraint stress induces mechanical and cold allodynia, and enhances inflammatory pain in rat: Relevance to human stress-associated painful pathologies. *Behav. Brain Res*. **205**, 360-6 (2009).

Blasco-Serra, A., *et al*. Depressive-like symptoms in a reserpine-induced model of fibromyalgia in rats. *Physiol. Behav*. **151**, 456-62 (2015).

Bonaterra, G.A., *et al*. Morphological alterations in gastrocnemius and soleus muscles in male and female mice in a fibromyalgia model. *PLoS One*. **11**, e0151116 (2016).

Carneiro de Oliveira, P.E., Zaniboni, C.R., Carmona, I.M., Fonseca, A.R. & Canto-de-Souza, A. Preliminary behavioral assessment of cagemates living with conspecifics submitted to chronic restraint stress in mice. *Neurosci. Lett.* **657**, 204-10 (2017).

Chen, W.K., *et al.* Ca(v)3.2 T-type Ca2+ channel-dependent activation of ERK in paraventricular thalamus modulates acid-induced chronic muscle pain. *J. Neurosci*. **30**, 10360-8 (2010).

de Freitas, C.M., *et al.* Behavioral and neurochemical effects induced by reserpine in mice. *Psychopharmacology (Berl).* **233**, 457-67 (2016).

De la Luz-Cuellar, Y.E., *et al.* Blockade of spinal α5-GABAA receptors differentially reduces reserpine-induced fibromyalgia-type pain in female rats. *Eur. J. Pharmacol*. **858**, 172443 (2019).

de Souza, A.H., *et al*. The effects of Phα1β, a spider toxin, calcium channel blocker, in a mouse fibromyalgia model. *Toxicon*. **81**, 37-42 (2014).

Dina, O.A., Green, P.G. & Levine, J.D. Role of interleukin-6 in chronic muscle hyperalgesic priming. *Neuroscience*. **152**, 521-5 (2008).

Ferrari, L.F., Araldi, D. & Levine, J.D. Distinct terminal and cell body mechanisms in the nociceptor mediate hyperalgesic priming. *J. Neurosci*. **35**, 6107-16 (2015).

Gameiro, G.H., *et al.* The effects of restraint stress on nociceptive responses induced by formalin injected in rat's TMJ. *Pharmacol. Biochem. Behav*. **282**, 338-44 (2005).

Green, P.G., Alvarez, P., Gear, R.W., Mendoza, D. & Levine, J.D. Further validation of a model of fibromyalgia syndrome in the rat. *J. Pain*. **12**, 811-8 (2011).

Guevara, C., Fernandez, A.C., Cardenas, R. & Suarez-Roca, H. Reduction of spinal PGE2 concentrations prevents swim stress-induced thermal hyperalgesia. *Neurosci. Lett.* **591**, 110-4 (2015).

Hernandez-Leon, A., De la Luz-Cuellar, Y.E., Granados-Soto, V., González-Trujano, M.E. & Fernández-Guasti, A. Sex differences and estradiol involvement in hyperalgesia and allodynia in an experimental model of fibromyalgia. *Horm. Behav*. **97**, 39-46 (2018).

Hsiao, I.H. & Lin, Y.W. Electroacupuncture reduces fibromyalgia pain by attenuating the HMGB1, S100B, and TRPV1 signalling pathways in the mouse brain. *Evid. Based. Complement. Alternat. Med.* **2022**, 2242074 (2022).

Huang, N., *et al.* Alterations in the BDNF-mTOR signaling pathway in the spinal cord contribute to hyperalgesia in a rodent model of chronic restraint stress. *Neuroscience*. **409**, 142-51 (2019).

Huang, Y., *et al.* Nitric oxide in the spinal cord is involved in the hyperalgesia induced by tetrahydrobiopterin in chronic restraint stress rats. *Front. Neurosci*. **15**, 593654 (2021).

Hung, C.H., *et al*. Activation of acid-sensing ion channel 3 by lysophosphatidylcholine 16:0 mediates psychological stress-induced fibromyalgia-like pain. *Ann. Rheum. Dis*. **79**, 1644-56 (2020).

Imbe, H., Kimura, A., Donishi, T. & Kaneoke, Y. Chronic restraint stress decreases glial fibrillary acidic protein and glutamate transporter in the periaqueductal gray matter. *Neuroscience*. **223**, 209-18 (2012).

Imbe, H., Kimura, A., Donishi, T. & Kaneoke, Y. Effects of restraint stress on glial activity in the rostral ventromedial medulla. *Neuroscience*. **241**, 10-21 (2013).

Inyang, K.E., *et al.* Alleviation of paclitaxel-induced mechanical hypersensitivity and hyperalgesic priming with AMPK activators in male and female mice. *Neurobiol. Pain*. **6**, 100037 (2019).

Jasper, L.L. & MacNeil, B.J. Diverse sensory inputs permit priming in the acidic saline model of hyperalgesia. *Eur. J. Pain*. **16**, 966-73 (2012).

Ji, Y., Hu, B., Li, J. & Traub, R.J. Opposing roles of estradiol and testosterone on stress-induced visceral hypersensitivity in rats. *J. Pain.* **19**, 764-76 (2018).

Kaneko, K., *et al*. The analgesic effect of tramadol in animal models of neuropathic pain and fibromyalgia. *Neurosci. Lett.* **562**, 28-33 (2014).

Khasar, S.G., Dina, O.A., Green, P.G. & Levine, J.D. Sound stress-induced long-term enhancement of mechanical hyperalgesia in rats is maintained by sympathoadrenal catecholamines. *J. Pain*. **10**, 1073-7 (2009).

Khasar, S.G., *et al.* Stress induces a switch of intracellular signaling in sensory neurons in a model of generalized pain. *J. Neurosci.* **28**, 5721-30 (2008).

Khasar, S.G., Green, P.G. & Levine, J.D. Repeated sound stress enhances inflammatory pain in the rat. *Pain*. **116**, 79-86 (2005).

Ledeboer, A, *et al.* Spinal cord glia and interleukin-1 do not appear to mediate persistent allodynia induced by intramuscular acidic saline in rats. *J. Pain*. **7**, 757-67 (2006).

Lee, H., *et al.* Effects of tianeptine on symptoms of fibromyalgia via BDNF signaling in a fibromyalgia animal model. *Korean J Physiol Pharmacol*. **21**, 361-70 (2017).

Liu, X., *et al.* Salvianolic acid B alleviates comorbid pain in depression induced by chronic restraint stress through inhibiting GABAergic neuron excitation via an ERK-CREB-BDNF axis-dependent mechanism. *J. Psychiatr. Res.* **151**, 205-16 (2022).

Liu, Y.T., Shao, Y.W., Yen, C.T. & Shaw, F.Z. Acid-induced hyperalgesia and anxio-depressive comorbidity in rats. *Physiol. Behav*. **131**, 105-10 (2014).

Lottering, B. & Lin, Y.W. Functional characterization of nociceptive mechanisms involved in fibromyalgia and electroacupuncture. *Brain Res.* **1755**, 147260 (2021).

Montserrat-de la Paz, S., García-Giménez, M.D., Ángel-Martín, M. & Fernández-Arche, A. Validation and additional support for an experimental animal model of fibromyalgia. *Mod. Rheumatol*. **25**, 116-22 (2015).

Mukae, T., Uchida, H. & Ueda, H. Donepezil reverses intermittent stress-induced generalized chronic pain syndrome in mice. *J. Pharmacol. Exp. Ther*. **353**, 471-9 (2015).

Murasawa, H., *et al.* Mirogabalin, a novel ligand for α2δ subunit of voltage-gated calcium channels, improves cognitive impairments in repeated intramuscular acidic saline injection model rats, an experimental model of fibromyalgia. *Biomed. Pharmacother*. **139**, 111647 (2021).

Nagakura, Y., *et al*. Different pathophysiology underlying animal models of fibromyalgia and neuropathic pain: comparison of reserpine-induced myalgia and chronic constriction injury rats. *Behav. Brain Res*. **226**, 242-9 (2012).

Nagakura, Y., *et al.* Monoamine system disruption induces functional somatic syndromes associated symptomatology in mice. *Physiol. Behav.* **194**, 505-514 (2018).

Nagakura, Y., *et al*. Spontaneous pain-associated facial expression and efficacy of clinically used drugs in the reserpine-induced rat model of fibromyalgia. *Eur. J. Pharmacol*. **864**, 172716 (2019).

Nagakura, Y., Oe, T., Aoki, T. & Matsuoka, N. Biogenic amine depletion causes chronic muscular pain and tactile allodynia accompanied by depression: A putative animal model of fibromyalgia. *Pain*. **146**, 26-33 (2009).

Nasu, T., Kubo, A., Queme, L.F. & Mizumura, K. A single administration of neurotropin reduced the elongated immobility time in the forced swimming test of rats exposed to repeated cold stress. *Behav. Pharmacol*. **30**, 547-54 (2019).

Nielsen, A.N., Mathiesen, C. & Blackburn-Munro, G. Pharmacological characterisation of acid-induced muscle allodynia in rats. *Eur. J. Pharmacol*. **487**, 93-103 (2004).

Nishiyori, M. & Ueda, H. Prolonged gabapentin analgesia in an experimental mouse model of fibromyalgia. *Mol. Pain*. **4**, 52 (2008).

Nishiyori, M., *et al.* Permanent relief from intermittent cold stress-induced fibromyalgia-like abnormal pain by repeated intrathecal administration of antidepressants. *Mol. Pain*. **7**, 69 (2011)

Nishiyori, M., Nagai, J., Nakazawa, T. & Ueda, H. Absence of morphine analgesia and its underlying descending serotonergic activation in an experimental mouse model of fibromyalgia. *Neurosci. Lett*. **472**, 184-7 (2010).

Oe, T., Tsukamoto, M. & Nagakura, Y. Reserpine causes biphasic nociceptive sensitivity alteration in conjunction with brain biogenic amine tones in rats. *Neuroscience*. **169**, 1860-71 (2010).

Paige, C., Maruthy, G.B., Mejia, G., Dussor, G. & Price, T. Spinal inhibition of P2XR or p38 signaling disrupts hyperalgesic priming in male, but not female, mice. *Neuroscience*. **2385**, 133-42 (2018).

Parada, C.A., Yeh, J.J., Reichling, D.B. & Levine, J.D. Transient attenuation of protein kinase C epsilon can terminate a chronic hyperalgesic state in the rat. *Neuroscience*. **120**, 219-26 (2003).

Peng, W.H., Kan, H.W. & Ho, Y.C. Periaqueductal gray is required for controlling chronic stress-induced depression-like behavior. *Biochem. Biophys. Res. Commun*. **593**, 28-34 (2022).

Quintero, L., Cardenas, R. & Suarez-Roca, H. Stress-induced hyperalgesia is associated with a reduced and delayed GABA inhibitory control that enhances post-synaptic NMDA receptor activation in the spinal cord. *Pain*. **152**, 1909-22 (2011).

Quintero, L., *et al.* Long-lasting delayed hyperalgesia after subchronic swim stress. *Pharmacol. Biochem. Behav*. **67**, 449-58 (2000).

Quintero, L., *et al.* Repeated swim stress increases pain-induced expression of c-Fos in the rat lumbar cord. *Brain Res.* **965**, 259-68 (2003).

Sharma, N.K., Ryals, J.M., Liu, H., Liu, W. & Wright, D.E. Acidic saline-induced primary and secondary mechanical hyperalgesia in mice. *J. Pain*. **10**, 1231-41 (2009).

Sikandar, S., *et al.* Brain-derived neurotrophic factor derived from sensory neurons plays a critical role in chronic pain. *Brain*. **141**, 1028-39 (2018).

Sluka, K.A., *et al.* Chronic hyperalgesia induced by repeated acid injections in muscle is abolished by the loss of ASIC3, but not ASIC1. *Pain*. **106**, 229-39 (2003).

Sluka, K.A., Kalra, A, & Moore, S.A. Unilateral intramuscular injections of acidic saline produce a bilateral, long-lasting hyperalgesia. *Muscle Nerve*. **24**, 37-46 (2001).

Suarez-Roca, H., *et al.* Role of mu-opioid and NMDA receptors in the development and maintenance of repeated swim stress-induced thermal hyperalgesia. *Behav. Brain Res.* **167**, 205-11 (2006).

Tanaka, M., *et al.* Establishment and assessment of a rat model of fatigue. *Neurosci. Lett*. **352**, 159-62 (2003).

Tillu, D.V., *et al.* Protease-activated receptor 2 activation is sufficient to induce the transition to a chronic pain state. *Pain*. **156**, 859-867 (2015).

Ueda, H. & Neyama, H. LPA1 receptor involvement in fibromyalgia-like pain induced by intermittent psychological stress, empathy. *Neurobiol. Pain*. **1**, 16-25 (2017).

Wakatsuki, K., *et al.* Peripheral nociceptive mechanisms in an experimental rat model of fibromyalgia induced by repeated cold stress. *Neurosci. Res*. **162**, 22-30 (2021).

Xu, G.Z., *et al.* Valproate reverses stress-induced somatic hyperalgesia and visceral hypersensitivity by up-regulating spinal 5-HT2C receptor expression in female rats. *Neuropharmacology*. **165**, 107926 (2020).

Yasui, M., *et al.* A chronic fatigue syndrome model demonstrates mechanical allodynia and muscular hyperalgesia via spinal microglial activation. *Glia*. **62**, 1407-17 (2014).

Yasui, M., *et al.* Hyperactivation of proprioceptors induces microglia-mediated long-lasting pain in a rat model of chronic fatigue syndrome. *J. Neuroinflammation*. **16**, 67 (2019).
